# Lewis superacids for catalytic reductions of stronger element–oxygen double bonds with hydrosilanes

**DOI:** 10.1039/d5qi02493e

**Published:** 2026-01-15

**Authors:** Daniel Franz, Thomas R. Frost, Sebastian Stigler, Shigeyoshi Inoue

**Affiliations:** a TUM School of Natural Sciences, Department of Chemistry, Catalysis Research Center and Institute of Silicon Chemistry, Technical University of Munich Garching bei München Germany s.inoue@tum.de

## Abstract

The main-group Lewis superacid complexes (pin^F^)_2_Si·MeCN (1·MeCN) and (pin^F^)_2_Ge·MeCN (2·MeCN) were successfully applied as promoters in the catalytic reduction of phosphine oxides (*e.g.*, Me_3_PO, Bu_3_PO, and Ph_3_PO), a sulfoxide (*i.e.*, Me_2_SO), and an amide (*i.e.*, Me_2_NCHO) to furnish the respective phosphines, dimethyl sulfide, and trimethylamine using silanes (*e.g.*, PhSiH_3_ and (EtO)_3_SiH) as hydrogen sources (pin^F^ = perfluoropinacolato). These substrates target difficult to reduce representatives of oxo compounds in comparison with, for example, the ketones or aldehydes often targeted in such types of catalytic reductions. As benchmark promoters, we also studied B(C_6_F_5_)_3_ and HNTf_2_ as reference (soft) Lewis superacid and Brønsted superacid, respectively (Tf = SO_2_CF_3_). Among the combinations of (pre)catalyst, substrate, and reducing agent investigated, the silicon complex **1**·MeCN turned out to be the most versatile system, being the by far most potent (DMSO) or just slightly underperforming (R_3_PO and DMF) promoter. Moreover, the hitherto undescribed Lewis acid base adducts 1·Me_2_NCHO and 2·Me_2_SO were synthesized, isolated, and structurally investigated using NMR spectroscopy and single-crystal XRD analysis.

## Abstract 2

Perfluoropinacolate complexes of Si and Ge possess Lewis superacid properties. They function as potent promoters in the reduction of highly stable P=O, S=O, and C=O double bonds with PhSiH_3_ and (EtO)_3_SiH.

## Graphical abstract

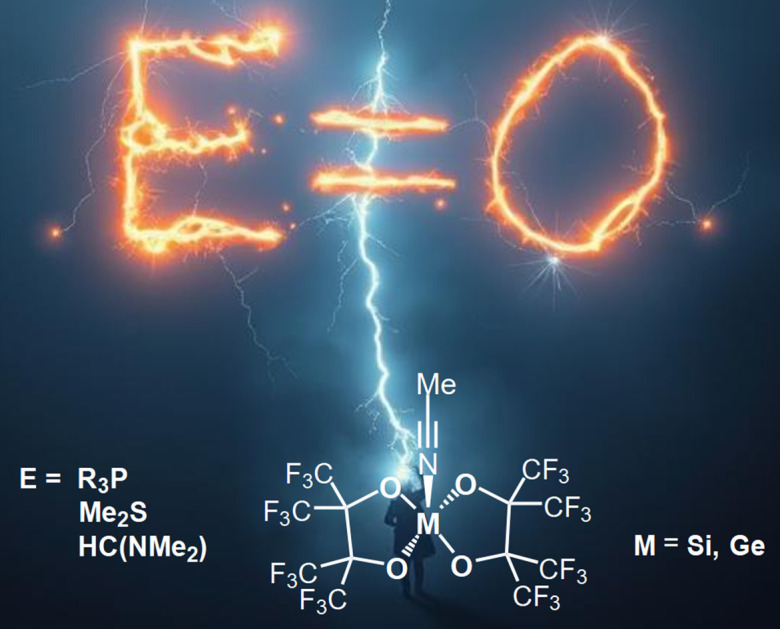

## Introduction

In the wake of Stephan's groundbreaking report on catalytic dihydrogenation with frustrated Lewis pairs and Power's pioneering article on the resemblance of low valent main-group elements with transition metals, the study of s- and p-block complexes for homogeneous catalysis markedly intensified.^1^ Stephan's, as well as Power's approaches, exploited the high reactivity profile of low-coordinate main group metal(loid) atoms. Similarly, the pronounced Lewis acidity of silyl cations derives not only from their coulombic attraction but also from their low-coordinated state. Consequently, silyl cations have evolved after Lambert's seminal finding in 1993 into a vast field of catalytic applications.^2^ As a more recent development, the use of strongly electron-withdrawing ligands was found to confer outstanding Lewis acidity to higher-coordinated and uncharged silicon complexes, as well, and the respective compounds were successfully applied in molecular catalysis.^3^ Notably, higher-coordinated cationic silicon complexes with outstanding Lewis acidity have also been reported.^4^

**Fig. 1:**
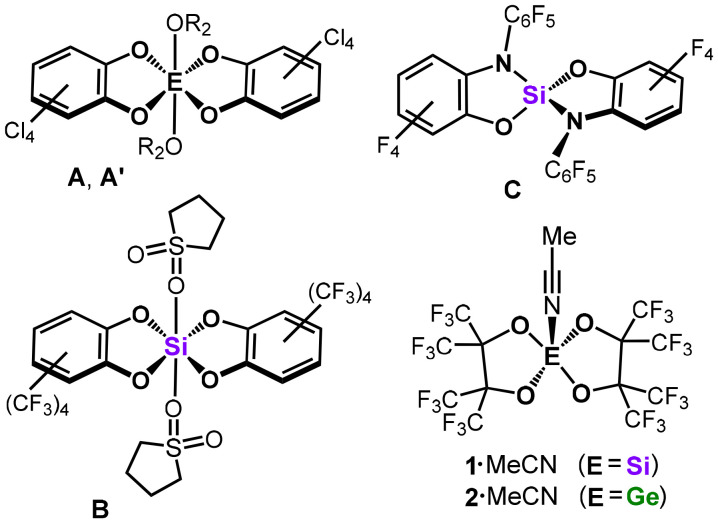
Selected group 14 Lewis acids. The biscatecholato complexes A, A′, and B, as well as the bis(*ortho*-amidophenolato) compound C and the bis(perfluoropinacolato) complexes 1·MeCN and 2·MeCN (A: E = Si, R = Et; A′: E = Ge, R = H).

For the classification of Lewis acids, Krossing coined the term ‘Lewis superacid’ as a category of complexes with a larger fluoride ion affinity (FIA) in the gas phase than antimony pentafluoride.^5^ Some controversy exists about the limitation of this threshold to a theoretical FIA, or whether experiment and theory need to coincide. More recently, Greb extended this concept to the definition of ‘soft Lewis superacids’, that is, molecular Lewis acids that have a larger hydride ion affinity (HIA) than B(C_6_F_5_)_3_ in the gas phase.^3*b*^ Perhalogenated species of the chelate fashioned catecholato ligand and its derivatives have been established as particularly suitable ligands for various Si and Ge complexes that meet the criteria for Lewis superacidity (selected species A–C, Fig. 1).^6^ In 2021, we reported the silicon complex **1**·MeCN, which bears the bidentate and strongly electron-withdrawing perfluoropinacolato ligand and abstracts fluoride from AgSbF_6_ in acetonitrile solution (Fig. 1).^7^ Also, the heavier germanium congener 2·MeCN was described, which exhibited larger FIA and HIA values (Fig. 1).^8^ These compounds promoted catalytic conversions such as hydrodefluorination and hydrosilylation (*i.e.*, reduction) of double bonds, as well as polyether degradation.^7–9^ In a detailed fashion we had investigated the catalytic hydrosilylation (*i.e.*, reduction) of ketones and aldehydes with 1·MeCN.^7^ In this work, we focus on more difficult to reduce element oxygen double bonds as found in phosphine oxides, sulfoxides, and amides using 1·MeCN and 2·MeCN as promoters. Moreover, we compare the catalytic activities of these with the ubiquitous benchmark Lewis acid B(C_6_F_5_)_3_ and the strong Brønsted acid HNTf_2_ (Tf = SO_2_CF_3_).

## Results and discussion

### Phosphine oxide reductions

**Fig. 2:**
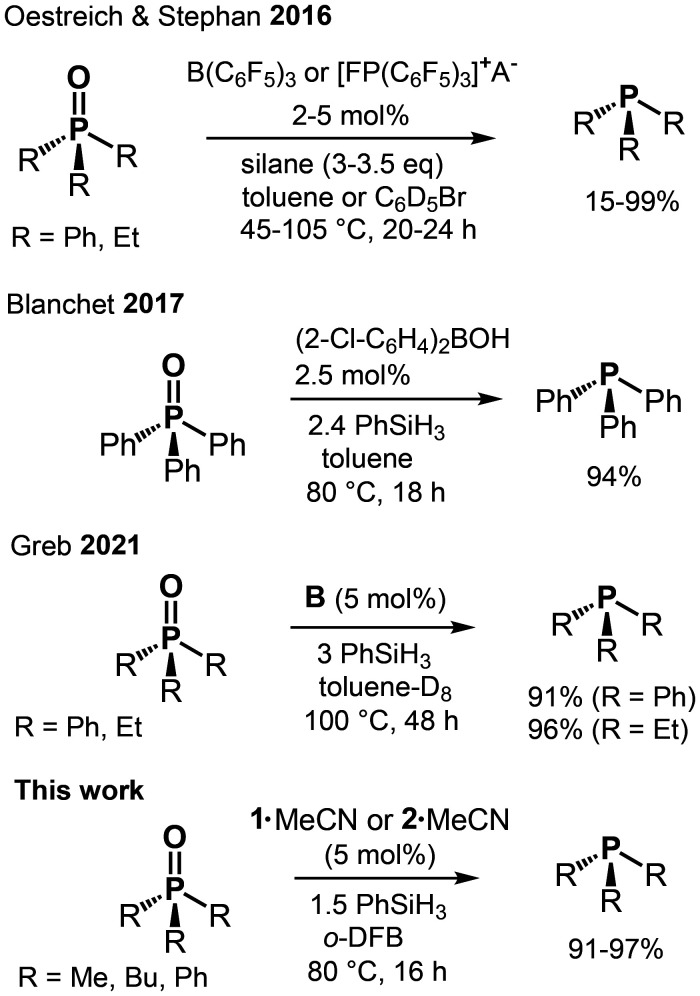
Selected catalytic tertiary phosphine oxide reductions to phosphine using main group complexes (A^−^ = B(C_6_F_5_)_4_^−^; silane = PhSiH_3_, (EtO)_3_SiH; *o*-DFB = *ortho*-difluorobenzene).

Phosphines are used in many synthetic applications because the formation of the strong phosphorus–oxygen double bond often drives a reaction, as seen in the Wittig and Mitsunobu reactions.^10^ The reduction of phosphine oxides back to the corresponding phosphines is attractive in the light of waste material recycling. Catalytic amounts of titanium(iv) alkoxy compounds were reported by Lawrence to catalyze the reduction of tertiary phosphine oxides by silanes.^11^ Beller used copper halides and copper(ii) triflate to facilitate the reduction of secondary and tertiary phosphine oxides with organosilanes.^12^ Notably, non-catalytic methods for the reduction of tertiary phosphine oxides using highly reactive silanes (*e.g.*, PhSiH_3_, Cl_3_SiH, and Si_2_Cl_6_) were reported about 60 years ago and commonly required harsh reaction conditions.^13^ Other procedures that work without a catalyst typically implement hydroboranes or aluminum hydrides and the reader is referred to the respective reviews for details.^14^ Prominent examples for phosphine oxide reduction by main-group promoters rely on potent Lewis acids of boron (*e.g.*, B(C_6_F_5_)_3_, (2-Cl-C_6_H_4_)_2_BOH) or highly Lewis acidic phosphonium cations (Fig. 2, rows 1 and 2).^15^ More recently, Greb described the implementation of the silicon Lewis superacid B in the reduction of Et_3_PO and Ph_3_PO at 100 °C in toluene using 3 eq. of PhSiH_3_ as a reducing agent (Fig. 2, row 3).^6*c*^

Notably, the experimental assessment of Lewis acidity is commonly conducted by the use of phosphine oxides and ^31^P NMR analysis, that is, the Gutmann–Beckett method.^16^ Accordingly, many Lewis superacids have been probed for complexation of Et_3_PO, but the further conversion of these complexes, if formed, has often not been investigated.

In the light of Greb's result, we set out to probe the catalytic activity of 1·MeCN and 2·MeCN for phosphine oxide reduction using Bu_3_PO, Me_3_PO, and Ph_3_PO as substrates. The ubiquitous Et_3_PO was left out due to its higher pricing and lower convenience (*i.e.*, waxy nature of the solid) which renders it less suitable for large-scale applications. As expected, the use of PhSiH_3_ resulted in the production of phosphine and the results of our catalytic conversions are shown in Table 1. The conversion of Bu_3_PO with 1.5 eq. of PhSiH_3_ and 1·MeCN or 2·MeCN as a promoter (5 mol%) furnished the respective phosphine in a near-quantitative fashion (97% or 96%) after 16 h in *ortho*-difluorobenzene (*o*-DFB) at 80 °C (Table 1, entries 1 and 2). Decreasing the catalyst load to 1 mol% 2·MeCN resulted in a markedly lower yield (75%), which might possibly be compensated by a longer reaction time (Table 1, entry 3). The alternative reducing agents Et_3_SiH (5.5 eq.) and pinacolborane (HBpin, 4.5 eq.) were also tested and resulted in practically no formation of phosphine (Et_3_SiH) and high phosphine yield (HBpin, 89%), respectively, which agrees with the expected deoxyhydrogenation activity of these compounds (Table 1, entries 4 and 5). In the context of applying the germanium complex 2·MeCN, we had described its reaction to a germylene species of the type pin^F^Ge (pin^F^ = ((CF_3_)_2_CO)_2_) upon reaction with Et_3_SiH.^8^ Though this reactivity is mostly favored in the absence of electron–pair donors, it may gain relevance at elevated temperature and in the presence of excess hydrosilane. Thus, we tested the germylene adduct (pin^F^Ge)_2_·(1,4-dioxane) ((3)_2_·diox) as a promoter in catalytic phosphine oxide reduction, as well, after we had synthesized it independently as described in the literature.^8^ In fact, the use of (3)_2_·diox (5 mol% loading in Ge) afforded markedly lower yield (52%) of Bu_3_P in combination with PhSiH_3_ (1.5 eq.) in comparison with the reactions with 1·MeCN and 2·MeCN (Table 1, entries 6, 7, *cf.* entries 1 and 2). We assume that the reduced Lewis acidity of the germylene, as compared to the germane, accounts for the decreased activity of the former. Accordingly, the partial conversion of 2·donor into 3·donor might hamper the catalytic performance of **2**·MeCN in the course of the reaction and come into effect for longer reaction times or lower catalyst loadings, as we had observed (Table 1, entry 3). For comparing our Lewis acids with a strong Brønsted acid, we used HN(SO_2_CF_3_)_2_ as a (pre)catalyst, and the reduction of Bu_3_PO proceeded similarly to that of our Si and Ge promoters (Table 1, entry 8). One must note that in the absence of any promoter, the phosphine oxide was also reduced to a non-negligible degree (20%, Table 1, entry 9), which agrees with the literature reports.^17^ We also probed Me_3_PO as the substrate to find complete consumption of the oxide and formation of Me_3_P (91%, the non-quantitative detected yield is attributed to partial loss of volatile Me_3_P to the headspace) after just 5 h at 80 °C and, thus, considerably shorter than the *ca.* 16 h required for the reduction of the bulkier Bu_3_PO under very similar conditions (Table 1, entry 10 *vs.* 1). In stark contrast, Ph_3_PO was more difficult to reduce as we found only 69% of the respective phosphine after the full 16 h of reaction period (Table 1, entry 11). For comparison, Oestreich and Stephan reported near-quantitative conversion of Ph_3_PO with the use of B(C_6_F_5_)_3_ (5 mol%) in toluene at 105 °C over 20 h.^15*a*^

**Table 1:** Catalytic reduction of phosphine oxides to phosphines

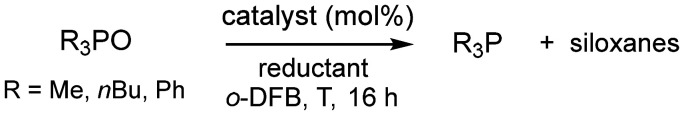					
	R	Cat. (mol%)	Red. (eq.)	*T* [°C]	Yield^a^ [%]
1	*n*Bu	1·MeCN (5)	PhSiH_3_ (1.5)	80	97
2	*n*Bu	2·MeCN (5)	PhSiH_3_ (1.5)	80	96
3	*n*Bu	2·MeCN (1)	PhSiH_3_ (1.5)	80	75
4	*n*Bu	2·MeCN (5)	Et_3_SiH (5.5)	110	<1
5	*n*Bu	2·MeCN (5)	HBpin (4.5)	80	89
6	*n*Bu	(3)_2_·diox (2.5)	PhSiH_3_ (1.5)	80	52
7	*n*Bu	(3)_2_·diox (2.5)	Et_3_SiH (5)	120	<1
8	*n*Bu	HNTf_2_ (5)	PhSiH_3_ (1.5)	80	99
9	nBu	**None**	PhSiH_3_ (1.5)	80	20
10	Me	1·MeCN (5)	PhSiH_3_ (1.5)	80	91^b^
11	Ph	2·MeCN (5)	PhSiH_3_ (1.5)	80	69

a Yield determined using ^31^P{^1^H} NMR by the addition of tris(2,4-di-*tert*-butylphenyl) phosphite as an internal standard (after the conversion).

b Yield determined using ^1^H NMR by the addition of 4,4′-di-*tert*-butyl-biphenyl as an internal standard (at conversion start).

### Dimethylsulfoxide reductions

**Fig. 3:**
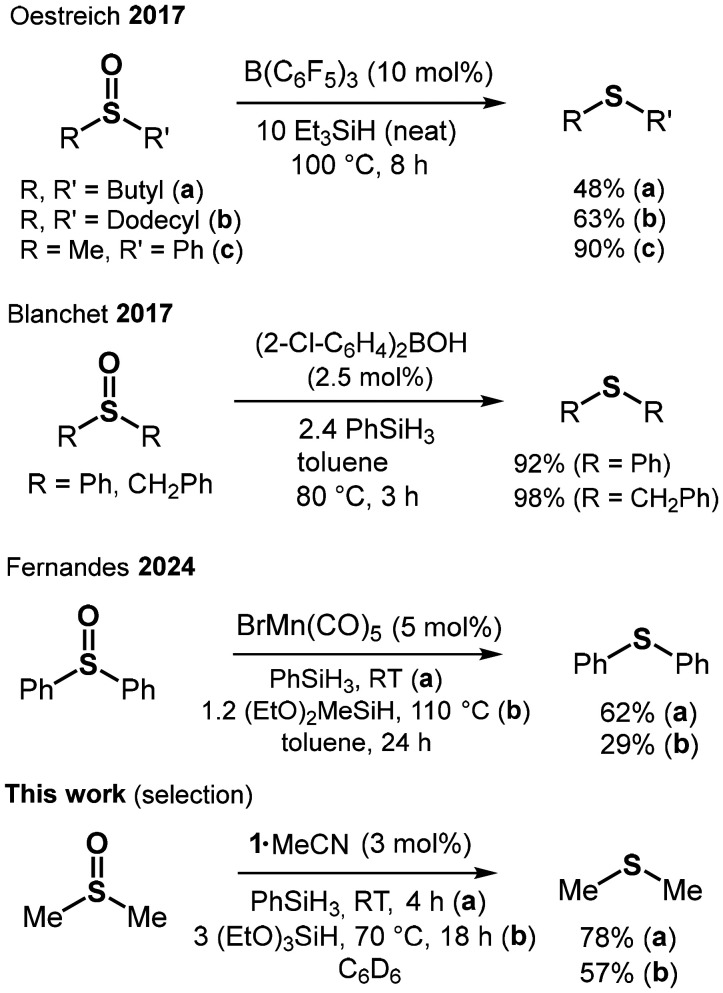
Selected catalytic reductions of sulfoxides to sulfides using hydrosilane reducing agents (RT = room temperature).

With our successful reduction of the P=O double bond as a starting point, we set out to apply similar conditions to the reduction of S=O double bonds. Me_2_SO (DMSO) marks one of the most widespread sulfoxides due to its application as a polar-aprotic solvent. Generally, sulfur possesses a higher electronegativity and a smaller atomic radius in comparison with phosphorus. These attributes will affect bond polarization and π-interaction with oxygen and, in consequence, grant a lesser degree of zwitterionic character to the S=O double bond as compared to the P=O double bond. The generally higher electron affinity of sulfur over phosphorus should facilitate the reduction of formal oxidation state S(+IV) to S(+II) as compared to the reduction of P(+V) to P(+III). The conversion of sulfoxides to sulfides is a vast field employing transition metal catalysis,^18^ main group catalysis,^15*b*,19^ electrochemical procedures,^20^ photochemical,^21^ and catalyst-free methods (Fig. 3).^22^ DMSO is one of the most fundamental sulfoxides and, due to its occurrence in the biosphere, the DMSO/DMS redox system plays an important role in biochemistry, as well as environmental and food analytics. For example, reductions include the use of molybdenum-containing enzyme DMSO reductase^23^ or rhodium(iii) and molecular hydrogen.^24^ Trace analysis of DMSO in natural water after its reduction to DMS with NaBH_4_ has been described.^25^ The occurrence of DMS in beer brewing processes is notable, as well.^26^

We converted DMSO with PhSiH_3_ (1.5 eq.) under the addition of 1·MeCN as a catalyst (5 mol%) in C_6_D_6_ at room temperature (RT). As a result, we observed full consumption of the DMSO and major conversion to Me_2_S (DMS, 73%) within 2 hours (using ^1^H and ^13^C NMR analysis with 4,4′-di-*tert*-butyl-biphenyl as the internal standard, Table 2, entry 1). This contrasts the increased temperature (80 °C) and longer reaction time (5 h) required for the reduction of Me_3_PO with PhSiH_3_. The discrepancy between the converted DMSO (>99%) and the determined DMS yield (73%) we attribute to a larger relevance of side-reactions for the conversion of this substrate as compared to the phosphine oxide reductions. As expected, decreasing the load of 1·MeCN and the equivalents of PhSiH_3_ resulted in lower conversion rates (Table 2, entries 2, 3 and 4). For the phosphine oxide reduction, the use of 2·MeCN as a (pre)catalyst had afforded just marginally less efficient conversions of the substrates. Remarkably, the catalytic DMSO reduction with PhSiH_3_ showed a substantially diminished performance at RT when using this germanium complex as a promoter (Table 2, entries 5 and 6). Using catalytic amounts of the preformed DMSO complex 2·Me_2_SO (see the supplementary information for its isolation) instead of 2·MeCN had a negligible effect on the conversion rate (Table 2, entry 7). It is reasonable to assume that a ‘softer’ sulfur atom has a higher affinity to the Ge center in 2 as it has to the ‘harder’ Si center in 1. Thus, 2·MeCN will suffer more strongly from ‘catalyst poisoning’ by the sterically unhindered product Me_2_S. We also employed both the reference acids B(C_6_F_5_)_3_ and HNTf_2_ as (pre)catalysts, which in resemblance to 2·MeCN, turned out to perform substantially worse than the silicon Lewis acid 1·MeCN (Table 2, entries 8 and 9). Notably, when no promoter was applied, 14% DMSO was consumed to yield 8% DMS at 100 °C over 24 h (Table 2, entry 10). Moreover, we investigated the use of Et_3_SiH and (EtO)_3_SiH as alternative reducing agents. These are less atom economical but commonly cheaper and more suitable for process upscaling than PhSiH_3_. The Et_3_SiH reducing agent (4 eq.) proved ineffective in our hands: very harsh conditions (100 °C, 24 h) provided only 6% yield in DMS (28% conversion of DMSO) when applying the potent 1·MeCN as a (pre)catalyst (Table 2, entry 11). In contrast, (EtO)_3_SiH (4 eq.) afforded conversion to DMS in a moderate yield (52%) at RT in C_6_D_6_ using 1·MeCN (5 mol%), though on a notably longer timescale (72 h, Table 2, entry 12). Changing the solvent (C_6_D_6_ for CDCl_3_) and lowering the equivalents of hydrides had only a minor impact on the reaction outcome (Table 2, entry 13). Conducting the catalysis at 70 °C in C_6_D_6_ boosted the conversion rate with only a negligible impact on product distribution (Table 2, entry 14). In resemblance to our finding with PhSiH_3_, three equivalents of hydride were required for an effective reduction: the use of only 2 eq. (EtO)_3_SiH resulted in incomplete DMSO conversion (70%) to afford 39% DMS at 70 °C (3 mol% load with 1·MeCN) even at prolonged reaction times (Table 2, entry 15). The high performance of 1·MeCN as a promoter was verified by decreasing the load down to 1 mol%, which delivered almost 2/3 of the DMS yield after the same reaction time as with 5 mol% load (Table 2, entry 16 *vs.* entry 12). With (EtO)_3_SiH as a reducing agent, the use of germanium complex 2·MeCN as a (pre)catalyst was, yet again, less effective than using the silicon Lewis acid (Table 2, entries 17 and 18). The Brønsted superacid promoter HN(Tf)_2_ was also markedly less effective than 1·MeCN (Table 2, entries 19 and 20). Notably, we observed the separation of oil and solid from the reaction solution. We found the combination of (EtO)_3_SiH with B(C_6_F_5_)_3_ as a (pre)catalyst least suitable: with a load of 5 mol% and use of 4 eq. silane we could not observe DMS formation even after allowing the experiment to run a couple of days beyond the 72 h time mark (Table 2, entry 21). The outcome that the boron Lewis acid does not promote the DMSO reduction in combination with (EtO)_3_SiH (at RT) but performs moderately when brought together with PhSiH_3_ needs to be considered in the light of the respective ^11^B NMR data: the ^11^B NMR spectrum of the triethoxysilane conversion reveals a singlet at −0.4 ppm (*h*_1/2_ = 230 Hz), which we ascribe to [EtOB(C_6_F_6_)_3_]^−^, though Me_2_SB(C_6_F_5_)_3_ is a conceivable species, as well.^27^ The ^11^B analysis of the PhSiH_3_ reaction similarly shows a major singlet at −0.2 ppm (*h*_1/2_ = 240 Hz) but also reveals a doublet at −24.7 ppm (*J* = 80 Hz) of minor intensity (see Fig. S21). The doublet can be assigned to the [HB(C_6_F_5_)_3_]^−^ anion, which was reported for catalytic conversions using combinations of hydrosilane and tris(pentafluorophenyl)borane and, of course, for catalytic dihydrogenations with frustrated Lewis pairs containing this borane Lewis acid.^28^ The borohydride anion may indicate the formation of highly Lewis acidic silyl cation or the borohydride itself may act as a hydride transfer reagent. Finally, it is of note that without the application of a catalyst, no relevant consumption of DMSO was indicated by the ^1^H NMR analysis at 100 °C for 24 h using 5 eq. of (EtO)_3_SiH (Table 2, entry 22).

**Table 2:** Catalytic reduction of DMSO to DMS using silanes

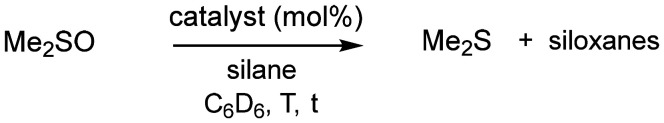					
	Cat. (mol%)	Silane (eq.)	*T*	*t*	Yield^a^ [%]
1	1·MeCN (5)	PhSiH_3_ (1.5)	RT	2 h	73
2	1·MeCN (3)	PhSiH_3_ (1)	RT	4 h	78
3	1·MeCN (1)	PhSiH_3_ (1)	RT	4 h	53^b^
4	1·MeCN (3)	PhSiH_3_ (^2^/_3_)	RT	24 h	66^c^
5	2·MeCN (5)	PhSiH_3_ (1.5)	RT	3 h	9
6	2·MeCN (3)	PhSiH_3_ (1)	RT	4 h	6
7	2·DMSO (3)	PhSiH_3_ (1)	RT	4 h	12^d^
8	B(C_6_F_5_)_3_ (5)	PhSiH_3_ (1)	RT	18 h	13
9	HNTf_2_ (5)	PhSiH_3_ (1.5)	RT	2 h	12^e^
10	**None**	PhSiH_3_ (3)	100 °C	24 h	8
11	1·MeCN (5)	Et_3_SiH (4)	100 °C	24 h	6
12	1·MeCN (5)	(EtO)_3_SiH (4)	RT	72 h	52^f^
13	1·MeCN (5)	(EtO)_3_SiH (3)	RT	72 h	40^g^
14	1·MeCN (3)	(EtO)_3_SiH (3)	70 °C	18 h	57
15	1·MeCN (3)	(EtO)_3_SiH (2)	70 °C	36 h	39^h^
16	1·MeCN (1)	(EtO)_3_SiH (4)	RT	72 h	31
17	2·MeCN (5)	(EtO)_3_SiH (4)	RT	72 h	26
18	2·MeCN (3)	(EtO)_3_SiH (3)	70 °C	18 h	12
19	HNTf_2_ (5)	(EtO)_3_SiH (4)	RT	72 h	10^e^
20	HNTf_2_ (5)	(EtO)_3_SiH (3)	RT	72 h	11^i^
21	B(C_6_F_5_)_3_ (5)	(EtO)_3_SiH (4)	RT	72 h	<1
22	**None**	(EtO)_3_SiH (5)	100 °C	24 h	<1

a Yield of DMS was determined by addition of 1,4-di-*tert*-butyl-biphenyl as internal standard.

b DMSO was fully consumed and yield was increased after 12 h.

c Monitoring for additional 6 h resulted only in negligible change.

d DMSO was fully consumed and 78% yield achieved after 13 h at 70 °C.

e An oil separated from the mixture.

f After a total of 96 h, DMSO was fully consumed and 58% yield was achieved.

g CDCl_3_ was used as the solvent instead of C_6_D_6_.

h No further conversion by additional heating for 12 h.

i CDCl_3_ was used as the solvent and a solid separated after few hours.

### Dimethylformamide reductions

**Fig. 4:**
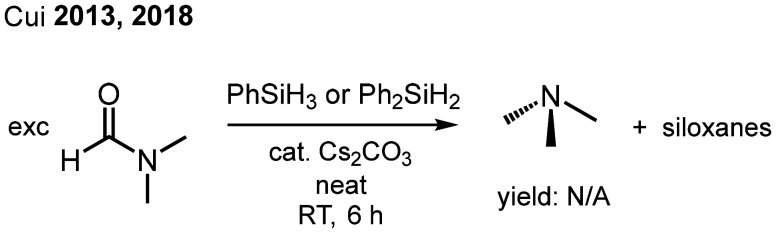
Reduction of DMF to Me_3_N as described by Cui.

DMF is difficult to reduce due to the delocalization of the nitrogen lone pair into the amide system, which renders the carbonyl carbon atom less electrophilic. It is used in large quantities as a solvent in the synthesis of peptides.^29^ DMF has a high boiling point (153 °C) and is commonly known for its hepatotoxicity.^30^ This combination of properties makes complete removal of the solvent from products most desirable, yet difficult. The ability to reduce DMF to volatile trimethylamine (boiling point 3 °C) would be beneficial for clean-up of reactions where large quantities of DMF waste are produced. Several methods for the catalytic reduction of DMF to Me_3_N by hydrosilanes using transition metal compounds as promoters have been described.^31^ Cui has reported the use of Cs_2_CO_3_ as a suitable catalyst for the reduction of DMF (and other amides) using phenylsilanes (Fig. 4).^32^

Given the potency of the Lewis acids 1·MeCN and 2·MeCN in the catalytic reduction of R_3_PO and Me_2_SO with silanes, we tested these systems for the transformation of DMF to Me_3_N using PhSiH_3_, as well. Promoting the reaction with 1·MeCN (5 mol%) in toluene-D_8_ furnished trimethylamine in a near-quantitative fashion (99%) at 110 °C over 12 h (Table 3, entry 1). Under milder reaction conditions (80 °C), a smaller ratio of Me_3_N was detected (71%) even after longer processing (24 h), which underlines the stability of this amide bond (Table 3, entry 2). The Ge complex 2·MeCN exhibited decreased catalytic activity (61%, Table 3, entry 3). The benchmark soft lewis superacid B(C_6_F_5_)_3_ performed similarly to 1·MeCN after 22 h (Table 3, entry 4). The ^11^B NMR spectra of the process revealed a signal at −24.7 ppm, which can be assigned to HB(C_6_F_5_)_3_, and this observation resembles the DMSO reduction with B(C_6_F_5_)_3_ and PhSiH_3_ described above. In the absence of a Lewis acid catalyst, no conversion of DMF was observed in toluene after 48 hours at 110 °C using 1.5 equivalents of PhSiH_3_ (Table 3, entry 5). To our knowledge, this is the first report of an uncharged tetrel Lewis superacid to successfully catalyze the reduction of an amide to an amine.

**Table 3:** Catalytic reduction of DMF to Me_3_N using silanes

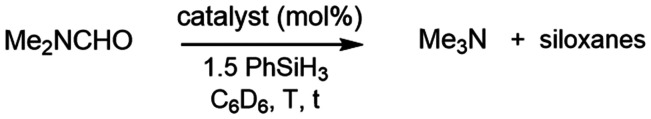					
	Cat. (mol%)	Solvent	*T* [°C]	*t*	Yield^a^ [%]
1	1·MeCN (5)	C_7_D_8_	110	12 h	99
2	1·MeCN (5)	C_6_D_6_	80	24 h	71
3	2·MeCN (5)	C_6_D_6_	80	24 h	61
4	B(C_6_F_5_)_3_ (5)	C_6_D_6_	80	22 h	70^b^
5	**None**	*o*-DFB	110	48 h	0

a Yield determined using ^1^H NMR with 4,4-di-*tert*-butyl-biphenyl as an internal standard.

b Near-quantitative consumption of DMF.

### Synthesis and isolation of 1·Me_2_NCHO and 2·Me_2_SO

**Scheme 1:**
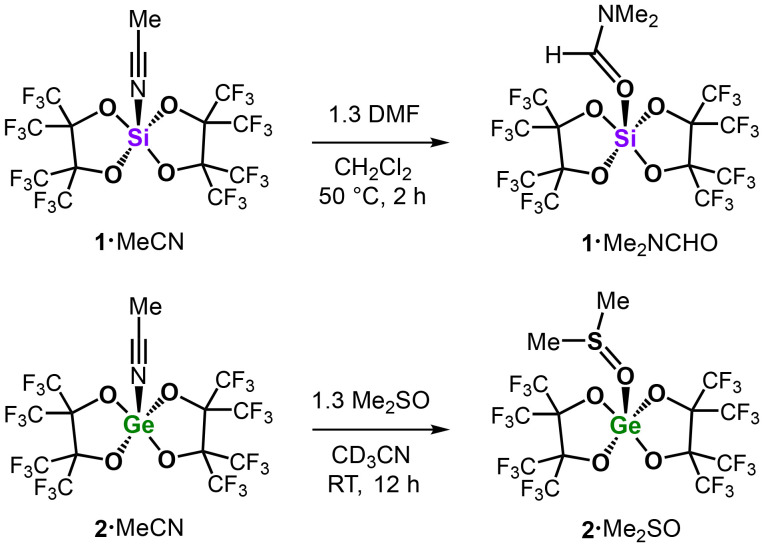
Synthesis of the tetrel complexes 1·Me_2_CH and 2·Me_2_SO.

In order to independently synthesize possible intermediates of our catalytic conversions, we reacted 1·MeCN and 2·MeCN with 1.3 eq. of DMF and DMSO, respectively (Scheme 1). The analytically pure Lewis acid base complexes 1·Me_2_NCHO and 2·Me_2_SO were isolated in good yields (89% and 84%) and characterized by NMR spectroscopy, combustion analysis (CHNS), mass spectrometry, and single-crystal XRD (SC-XRD) study.

**Fig. 5:**
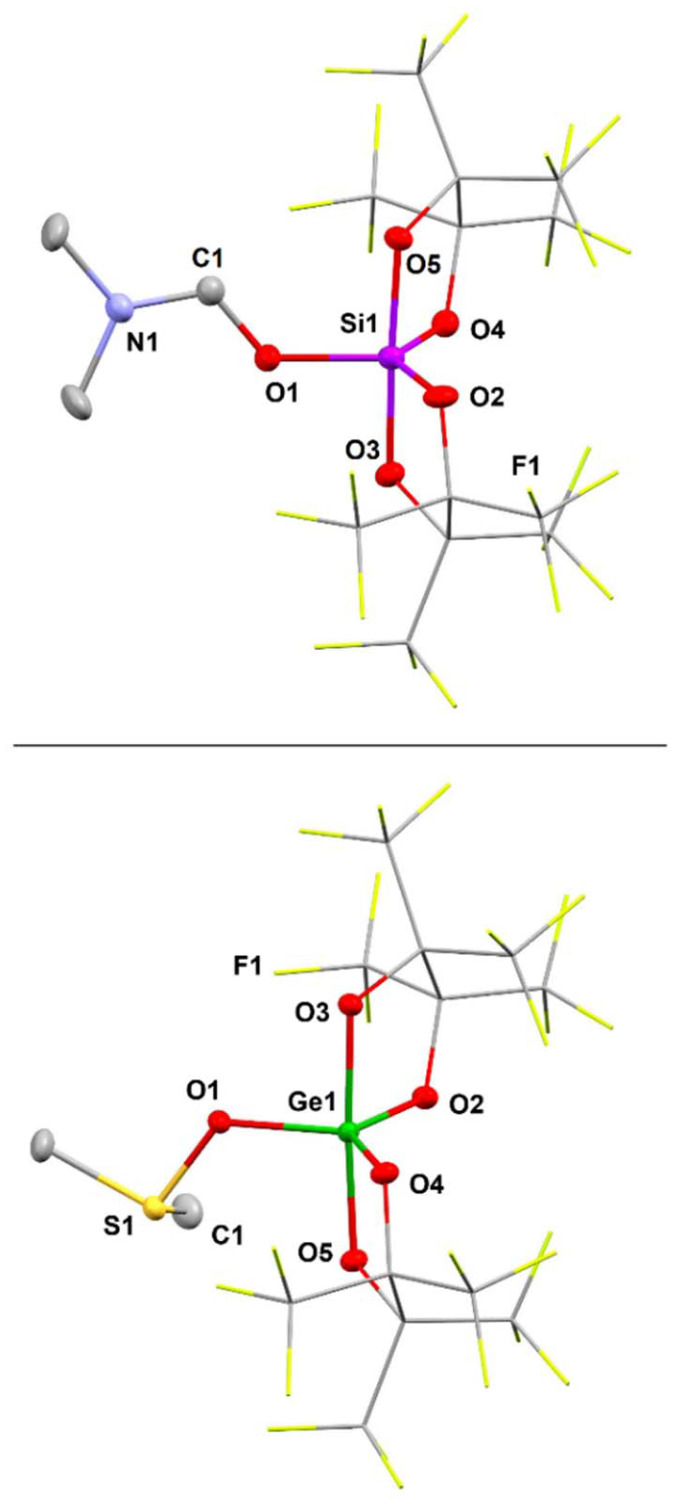
Molecular structure of 1·Me_2_NCHO (top) and 2·Me_2_SO (bottom) as ellipsoid plots (50% probability level) as derived from SC-XRD study. Hydrogen atoms are omitted for clarity. The (C(CF_3_)_2_)_2_ groups are displayed as capped sticks. One lattice MeCN is not shown (bottom). Selected structural parameters [Å, °]: Top: Si1–O1 = 1.718(2), Si1–O2 = 1.686(2), Si1–O3 = 1.743(2), Si1–O4 = 1.685(2), Si1–O5 = 1.730(2), C1–N1 = 1.289(5), C1–O1 = 1.296(4); O1–Si1–O2 = 117.9(1), O1–Si1–O4 = 112.9(1), O2–Si1–O4 = 129.1(1). Bottom: Ge1–O1 = 1.820(1), Ge1–O2 = 1.801(1), Ge1–O3 = 1.836(1), Ge1–O4 = 1.802(1), Ge1–O5 = 1.854(1), S1–O1 = 1.596(1); O1–Ge1–O2 = 113.5(1), O1–Ge1–O4 = 118.6(1), O2–Ge1–O4 = 127.9(1).

As a notable characteristic in the ^1^H NMR spectrum of 1·Me_2_NCHO the signals of the Si-coordinated DMF are notably shifted to lower fields (*δ* = 8.32, 3.33, and 3.18 ppm) as compared to “free” DMF in CD_3_CN.^33^ Suitable crystals of 1·Me_2_NCHO for SC-XRD analysis were obtained from a saturated CH_2_Cl_2_/MeCN (2 : 1) solution at −35 °C. The study reveals a silicon center that is coordinated in a trigonal bipyramidal fashion with the DMF ligand assuming an equatorial position (Fig. 5, top). The C=O bond length in 1·Me_2_NCHO amounts to C1–O1 = 1.296(4) Å, which is elongated as compared to DMF in the solid state (note: crystalline DMF forms a hydrogen bonding network with a mean C–O of 1.23 Å).^34^ This suggests a weakening in the C=O bond and may facilitate the hydride-induced reductive cleavage.

In the ^1^H NMR spectrum of 2·Me_2_SO, the singlet for the CH_3_ groups of coordinated DMSO at 3.10 ppm (*J*_^1^H^13^C_ = 144 Hz) is notable, which is markedly shifted to lower field as compared to the value for “free” Me_2_SO in CD_3_CN (2.50 ppm).^33^

Crystals of 2·Me_2_SO, suitable for SC-XRD analysis, were crystallized directly from the reaction mixture at −25 °C and the molecular structure of 2·Me_2_SO in the solid state marks a trigonal bipyramidal coordinate Ge center (sum of the equatorial bond angles ≈ 360°, Fig. 5, bottom). The bond to the oxygen atom of the (equatorial) DMSO ligand amounts to a length of Ge1–O1 = 1.820(1) Å. This distance is in between the values for the Ge–O distances involving the perfluoropinacol ligands, which show longer bonds for the axial positions (1.836(1) Å, 1.854(1) Å) and shorter bonds for the equatorial positions (1.801(1) Å, 1.802(1) Å). Notably, the S1–O1 distance of 1.596(1) Å is elongated relative to the respective bond lengths in Greb's octahedral coordinate (cat^Cl^)_2_Ge·(Me_2_SO)_2_ (Ge–O_DMSO_ = 1.561(1) Å, symmetry equivalent).^6*b*^ Interestingly, Greb's SC-XRD study also involves an uncoordinated lattice DMSO with a shorter S–O bond length of 1.502(1) Å that may serve as a reference for “free” Me_2_SO in the condensed phase. These data indicate that the S=O bond strength in 2·Me_2_SO is slightly lower than in (cat^Cl^)_2_Ge·(Me_2_SO)_2_. We assume that the six-coordinate Ge center in Greb's compound bearing two Me_2_SO donor ligands draws less electron density from each sulfoxide than the five-coordinate Ge center in 2·Me_2_SO, which bears only one Me_2_SO donor ligand to compensate its electron deficiency. Future studies might investigate into a correlation between the S–O distance in sulfoxide adducts of potent Lewis acids and their catalytic activity in sulfoxide reduction.

To test the relative affinity of the DMSO ligand to 2, we added DMF (2 eq.) to a CD_3_CN solution of **2**·Me_2_SO in an NMR sample tube, and ^1^H NMR analysis revealed a marked upfield shift of the CH_3_ signal of the DMSO protons. Notably, only one signal (set) for the DMSO (2.83 ppm), as well as the DMF (8.03, 3.00, 2.87 ppm) was observed neither of which corresponded to the respective ^1^H chemical shift of the “free” sulfoxide or amide, respectively (*δ*(^1^H) = 2.50 or 7.92, 2.89, and 2.77 ppm in CD_3_CN),^33^ but was consistently shifted to lower field. This suggests that DMSO and DMF are in a dynamic competitive equilibrium toward coordination to the Ge center in 2 (participation of CD_3_CN cannot be fully excluded), which is quick on the NMR timescale (at RT). The addition of Bu_3_PO (1.1 eq.) to this mixture resulted in a (further) upfield shift of the DMSO and DMF signals in the ^1^H NMR spectrum to values that match the “free” oxo compounds. In the ^31^P{^1^H} NMR analysis, one broad resonance at 87.2 ppm was found, which refers to the ^31^P signal observed, as also, upon mixing 2·MeCN and this phosphine oxide in CD_3_CN (without DMSO and DMF). We presume that Bu_3_PO majorly extrudes both DMSO and DMF from the Ge complex to furnish the more stable 2·Bu_3_PO. In addition, we converted 2·Me_2_SO with PhSiH_3_ (1.5 eq.) in CD_3_CN, which resulted in the anticipated formation of Me_2_S but also complete decomposition of 2 (and not refurnishing of 2·Do, Do = SMe_2_, CD_3_CN and Me_2_SO, as expected from a “true” catalyst) as concluded from ^1^H and ^19^F NMR analysis (full conversion after 9 h at 70 °C). This also shows that the Lewis acid-catalyzed reduction of DMSO to DMS with PhSiH_3_ can be conducted in MeCN, though conversion rates might be lower.

### Considerations in the catalysis mechanism

We have shown using NMR spectroscopy and SC-XRD study that 1 and 2 form strong coordination compounds with Bu_3_PO, Me_2_SO, and Me_2_NCHO. Complex 2·MeCN was reported to react with hydrosilane to the germylene species 3 and silylated perfluoropinacol and we showed that the latter exhibits weaker catalytic activity.^8^ In contrast, we found 1·MeCN not to react with PhSiH_3_ at RT (^1^H, ^19^F NMR monitored for 4 h in CD_3_CN; at 70 °C traces of new species noted after 2 h). Tilley proposed a Lewis acid catalysis mechanism for the reduction of aldehydes with Et_3_SiH using bis(perfluorocatecholato)silane as catalyst.^3*a*^ The catalytic cycle marks the initial interaction of the Lewis acid with the carbonyl group of the substrate. An alternative mechanism, which comprises an initial interaction between the Lewis acid and the hydrosilane (to effect weakening of the Si–H bond), was suggested by Oestreich and Stephan for the phosphine oxide reduction with silanes promoted by B(C_6_F_5_)_3_ or electrophilic phosphonium cations.^15*a*^ We reason that for the stronger adducts 1·Do (Do = R_3_PO, Me_2_SO, and Me_2_NCHO) the direct interaction of the Si center with an SiH group will be even more diminished than for 1·MeCN. Accordingly, we conclude that the catalytic reductions of the E=O double bonds (E = P, S, C) presented in this study proceed similar to Tilley's Lewis acid catalysis mechanism when 1·MeCN is used as the promoter. For 2·MeCN, the situation is more ambiguous due to its pronounced hydride affinity. Consistent to Tilley's mechanism we suggest that the respective complexes 1·Do or 2·Do (Do = R_3_PO, Me_2_SO, Me_2_NCHO) mark the actual catalysts which renders the MeCN adducts to assume the role of precatalysts. It is remarkable that **1**·MeCN seems to outperform the more Lewis acidic **B** in catalytic reduction of Ph_3_PO (Fig. 1, 2, and Table 1). Notably, strong Lewis acids of perhalogenated bis(catecholato) tetrelanes form hexacoordinate complexes with many Lewis bases, and, in stark contrast, the many bis(perfluoropinacol) tetrelanes which we have structurally characterized, so far, are limited to five-fold coordination. This demonstrates the pronounced impact of the ligand system (catecholate *vs.* pincacolate) on catalyst activity.

## Experimental

The relevant experimental work was conducted under an argon atmosphere using standard Schlenk techniques and a glovebox equipment. A general and representative procedure for the phosphine oxide reduction is as follows: an NMR sample tube was charged with the catalyst, and the phosphine oxide and the solids were dissolved in *o*-DFB. The reducing agent was added, and the reaction mixture was heated to the respective temperature for 16 hours. Tris(2,4-di-*tert*-butylphenyl) phosphite was added as an internal standard to determine the yield *via* intensity ratios in the ^31^P{^1^H} NMR spectrum. More detailed experimental data are given in the SI of this article.

## Conclusions

The main group Lewis acids 1·MeCN and 2·MeCN were successfully applied as precatalysts in the reduction of phosphine oxides (*e.g.* Me_3_PO, Bu_3_PO, and Ph_3_PO), a sulfoxide (*i.e.* Me_2_SO), and an amide (*i.e.* Me_2_NCHO) to afford the respective phosphines, dimethyl sulfide, and trimethylamine using PhSiH_3_ or (EtO)_3_SiH. These substrates mark generally more stable element oxygen double bonds in comparison with, for example, the C=O double bonds in ketones or aldehydes often targeted for demonstrating the catalytic activity of Lewis acids. As benchmarks, we also studied B(C_6_F_5_)_3_ and HNTf_2_ as reference (soft) Lewis superacid and Brønsted superacid, respectively. Among all the investigated combinations of (pre)catalyst, substrate, and reducing agent, we pronounce the silicon complex **1**·MeCN as the most versatile system, being the by far most potent (DMSO) or just slightly underperforming (R_3_PO, DMF) promoter. For the methylated substrates, we sort the ease of catalytic reduction using 1·MeCN and PhSiH_3_ in the order Me_2_SO > Me_3_PO > Me_2_NCHO (most facile to most difficult). Moreover, the hitherto undescribed Lewis acid base adducts 1·Me_2_NCHO and 2·Me_2_SO were synthesized, isolated, and structurally investigated using multinuclear NMR spectroscopy and single-crystal XRD analysis. After probing the reactivity of 1·MeCN, 2·MeCN, and **2**·Me_2_SO with DMF, DMSO, phosphine oxide, and PhSiH_3_, we conclude that a Lewis acid catalysis mechanism prevails as had been proposed by Tilley for silane Lewis acids. The MeCN complexes act as precatalysts to *in situ* form the catalytically active species 1·Do or 2·Do (Do = Me_3_PO, Me_2_SO, Me_2_NCHO). Future studies should focus on extending the scope of sulfoxides and amides, as well as suitable reducing agents. Other substrates such as esters should be investigated, and the water tolerance of the system needs to be examined.

## Author contributions

S. I. conceived and guided the study. D. F. and T. F. conceived and conducted the specific experiments. S. S. collected, solved, and refined the SC-XRD data. All authors have co-written the manuscript.

## Conflicts of interest

The authors declare no conflict of interest.

## Supplementary Material

QI-013-D5QI02493E-s001

QI-013-D5QI02493E-s002

## Data Availability

Supporting data for this article have been included as part of the supplementary information (SI). Supplementary information: detailed experimental and crystallographic data. See DOI: https://doi.org/10.1039/d5qi02493e. Further raw data are available upon reasonable request from the corresponding author. CCDC 2492264 and 2492265 contain the supplementary crystallographic data for this paper.^35*a*,*b*^
