# Supplementary material for: Lewis superacids for catalytic reductions of stronger element–oxygen double bonds with hydrosilanes
Source: Inorg Chem Front. 2026 Jan 15;13(7):2989–98. doi: 10.1039/d5qi02493e (PMC12857533; doi:10.1039/d5qi02493e)
Supplement: QI-013-D5QI02493E-s001 [file QI-013-D5QI02493E-s001.pdf]

Supplementary Information (SI) for Inorganic Chemistry Frontiers.
This journal is © the Partner Organisations 2026

Supporting Information

Lewis Superacids for Catalytic Reductions of Stronger Element-Oxygen Double Bonds with Hydrosilanes

D. Franz, T. R. Frost, S. Stigler, S. Inoue*

TUM School of Natural Sciences, Department of Chemistry, Catalysis Research Center and Institute of Silicon Chemistry

Technical University of Munich

Lichtenbergstraße 4, 85748 Garching bei München, Germany

E-Mail: s.inoue@tum.de

Table of Contents

1. General Considerations 2

1.1 General Information 2

1.2 Reagents and Solvents 2

1.3 Analysis 2

2. Synthesis and Catalysis 4

2.1 $(pin^F)_2Si{\cdot}Me_2NCHO$ ($1{\cdot}Me_2NCHO$) 4

2.2 $(pin^F)_2Ge{\cdot}Me_2SO$ ($2{\cdot}Me_2SO$) 10

2.3 Catalytic Reduction of Phosphine Oxides 20

2.4 Catalytic Reduction of DMSO 24

2.5 Catalytic Reduction of DMF 31

3. Crystallographic Data 34

4. References 37

1. General Considerations

1.1 General Information

Handling of air-sensitive compounds and reactions were conducted under inert argon 4.6 (≥ 99.996 % *Westfalen AG*) atmospheres in heat dried borosilicate Schlenk reaction vessels (applying standard Schlenk techniques) or in *LABstar Pro* gloveboxes from *MBraun Inertgas-Systeme GmbH* (unless stated otherwise). Glass junctions were coated with *Triboflon III* PTFE/PEPE grease. Any plastic or other heat sensitive apparatus, like syringes, were stored in original packaging and cycled three times with argon before use.

1.2 Reagents and Solvents

Any commercially available chemicals used for syntheses and reaction tests were purchased from commercial distributors: *Sigma-Aldrich, Merck (Darmstadt), Thermo Fischer,* and *TCI Deutschland, abcr, BLDpharm*. These were used as received.

Solvents were distilled over calcium hydride (CH_2Cl_2, *o*-DFB, acetonitrile), distilled over sodium/benzophenone (1,4-dioxane) or drawn from an MBraun Solvent Purification System (pentane, toluene) and stored over molecular sieves (3 Å) in Schlenk flasks or in a glovebox before using. Deuterated solvents (C_7D_8, C_6D_6, $CDCl_3$, CD_3CN) were dried and stored over molecular sieves (3 Å) in a glovebox.

1.3 Analysis

1.3.1 NMR Analysis

All NMR experiments were done in either *J. Young* PTFE tap NMR tubes or standard NMR tubes with a rubber cap that was supported by applying PTFE tape. All NMR spectra were recorded on an *Ascend 400* or *Ultrashield 400* spectrometers produced by *Bruker* at ambient temperature (298 K) unless stated otherwise.

δ-values are all stated in parts per million [ppm] and *J*-coupling constants are all stated in Hertz (Hz). In the assignment, abbreviations for multiplicity were used: singlet (s), doublet (d), triplet (t), quartet (q), quintet (qt), sextet (s), septet (sp), broad (br), and multiplet (m). The ^{1}H and ^{13}C spectra obtained were calibrated using the residual solvent peaks of the deuterated solvent as an internal standard as found in the literature.[S1] The data was analysed and processed using *MestReNova (version 14.3.0)*.

Any NMR experiments done in a non-deuterated solvent (*e.g. o*-DFB, toluene) contained a 'lock'- capillary filled with C_6D_6 to enable the shim/lock routine for well-resolved spectra.

1.3.2 Single Crystal X-ray Diffraction Analysis

The X-ray intensity data were collected on an X-ray single crystal diffractometer equipped with a CMOS detector (Bruker Photon-100), a rotating anode (Bruker TXS) with MoKα radiation (λ = 0.71073 Å) and a Helios mirror optic by using the APEX4 software package[S2] or an X-ray single crystal diffractometer equipped with a CMOS detector (Bruker Photon-

100), an IMS microsource with MoKα radiation (λ = 0.71073 Å) and a Helios mirror optic by using the APEX4 software package.[S2] The measurement was performed on single crystals coated with perfluorinated ether. The crystal was fixed on the top of a microsampler, transferred to the diffractometer and measured under a stream of cold nitrogen. A matrix scan was used to determine the initial lattice parameters. Reflections were merged and corrected for Lorenz and polarization effects, scan speed, and background using SAINT.[S3] Absorption corrections, including odd and even ordered spherical harmonics were performed using SADABS.[S4] Space group assignments were based upon systematic absences, E statistics, and successful refinement of the structures. Structures were solved by direct methods with the aid of successive difference Fourier maps, and were refined against all data using the APEX4[S2] in conjunction with SHELXL-2018/3.[S5,S6] and SHELXLE.[S7] Methyl hydrogen atoms were refined as part of rigid rotating groups, with a C–H distance of 0.98 Å and Uiso(H) = 1.5·Ueq(C). Other H atoms were placed in calculated positions and refined using a riding model, with methylene and aromatic C-H distances of 0.99 and 0.95 Å, respectively, and Uiso(H) = 1.2·Ueq(C). If not mentioned otherwise, non-hydrogen atoms were refined with anisotropic displacement parameters. Full-matrix least-squares refinements were carried out by minimizing $\Delta w(Fo^2\text{-}Fc^2)^2$ with SHELXL-2014[S8] weighting scheme. Neutral atom scattering factors for all atoms and anomalous dispersion corrections for the non-hydrogen atoms were taken from International Tables for Crystallography.[S9] Images of the crystal structures were generated by PLATON and MERCURY.[S10,S11] The CCDC numbers CCDC-2492264 and CCDC-2492265 contain the supplementary crystallographic data for the structures **1**·Me_2NHCO and **2**·Me_2SO. These data can be obtained free of charge from the Cambridge Crystallographic Data Centre via https://www.ccdc.cam.ac.uk/structures/.The crystallographic information files (CIF) were generated using FinalCif.[S12]

1.3.3 Mass Spectrometry Analysis

The mass spectrum was recorded using Liquid Injection Field Desorption Ionisation Mass Spectrometry (LIFDI-MS) and was measured directly from an inert atmosphere glovebox with a *Thermo-Fisher Scientific Exactive Plus Orbitrap* equipped with an ion source from *Linden CMS*.[S13] The samples were provided as filtered solutions in toluene.

2. Synthesis and Catalysis

Previous literature known compounds which are not commercially available were synthesised as described in the literature: $(pin^F)_2Ge\cdot MeCN$,[S14] and $[(pin^F)Ge]_2\cdot$1,4-dioxane.[S14] The synthesis of $(pin^F)_2Si\cdot MeCN$ was altered to use $SiBr_4$ as the silicon source instead of $SiCl_4$ because better yields were obtained with the bromide.[S15]

2.1 $(pin^F)_2Si\cdot Me_2NCHO$ (1·Me_2NCHO)

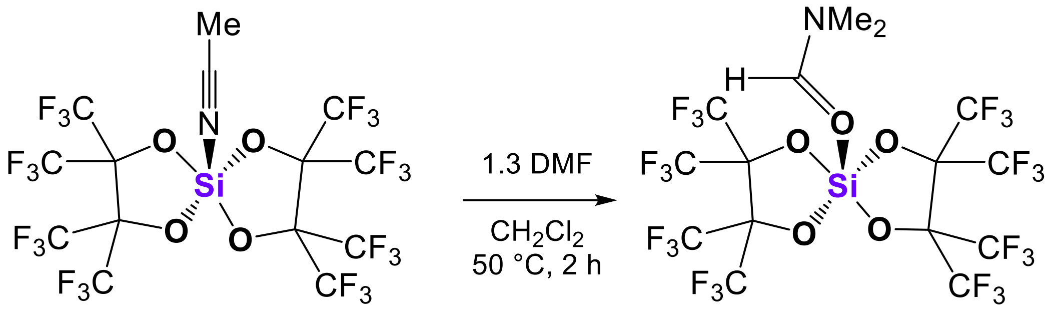

1·MeCN (0.241 g, 0.328 mmol, 1.0 eq.) was dissolved in CH_2Cl_2 (5.0 mL). DMF (0.031 g, 0.033 mL, 0.42 mmol, 1.3 eq.) was added. The reaction mixture was stirred at 50 °C for 2 h to ensure full solvation and subsequent reaction of **1**·MeCN. The crude product was washed thoroughly with pentane (5 mL, 1 h). This afforded **1**·Me_2NCHO (0.224 g, 0.293 mmol, 89%, crude) as a colourless powder. Traces of uncoordinated DMF were still present as verified by ^1H NMR spectroscopy. This additional DMF can be removed by sublimation in vacuum at elevated temperatures (verified by ^1H NMR spectroscopy) which might result in decreased yield. Elemental analysis was performed on the crude material.

X-ray quality crystals were obtained from a saturated CH_2Cl_2/MeCN (10:3) solution at −25 °C. For crystal structure please see Fig. S24, Section 3.

^1H NMR (400.2 MHz, CD_3CN, 298 K): δ = 8.3 (s, 1 H, CHO), 3.4 (s, 3 H, N(C*H*$_3$)(CH_3)), 3.2 (s, 3 H, N(CH_3)(C*H*$_3$))
$^{13}C\{^1H\}$ NMR (100.6 MHz, CD_3CN, 298 K): δ = 162.6 (CHO), 82.7 (br, O*C*(CF_3)$_2$), 41.1 (N(*C*H_3)(CH_3)), 35.9 (N(CH_3)(*C*H_3))
$^{13}C\{^1H,^{19}F\}$ NMR (100.6 MHz, CD_3CN, 298 K):): δ = 162.6 (CHO), 121.5 (CF_3), 82.6 (O*C*(CF_3)$_2$), 41.1 (N(*C*H_3)(CH_3)), 35.9 (N(CH_3)(*C*H_3))
^{19}F NMR (376.6 MHz, CD_3CN, 298 K): δ = −69.9 (m, 12 F, CF_3), −70.5 (m, 12 F, CF_3)
$^{29}Si\{^1H\}$ NMR (79.5 MHz, CD_3CN, 298 K): δ = −107.9 (Si)
Mass Spectrometry: Found = 765.97444 (LIFDI), calculated for $[C_{15}H_8F_{24}SiO_5N]^+$ = 765.97885
CHNS elemental analysis calcd [%] for $C_{15}H_7F_{24}SiO_5N$ [765.27]: C 23.73, H 0.80, N 1.96; found [%] C 23.54, H 0.92, N 1.83

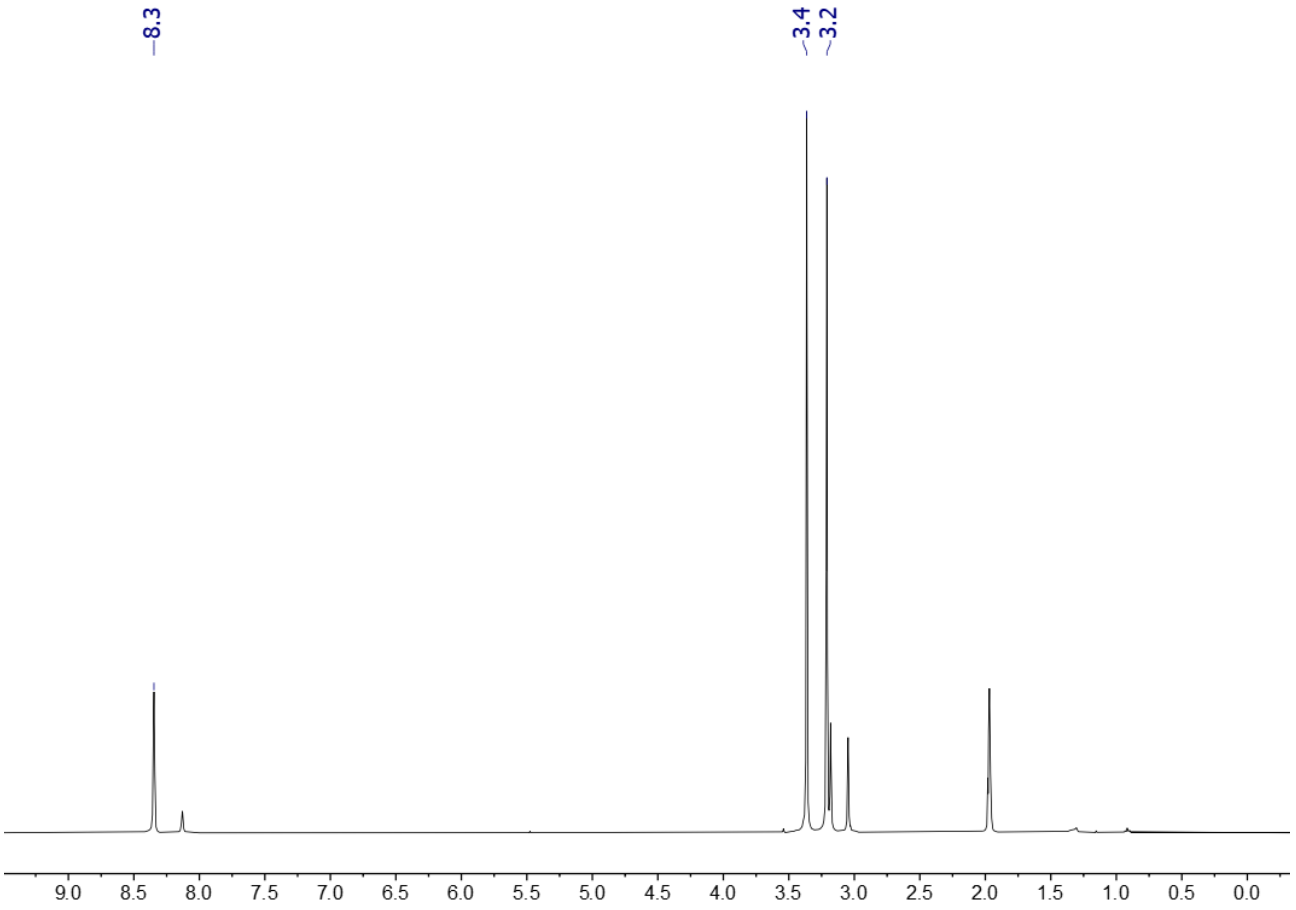

Fig. S1. ^{1}H NMR spectrum (400.2 MHz) of crude **1**·Me_2NCHO in CD_3CN. Note: the spectrum shows traces of uncoordinated DMF.

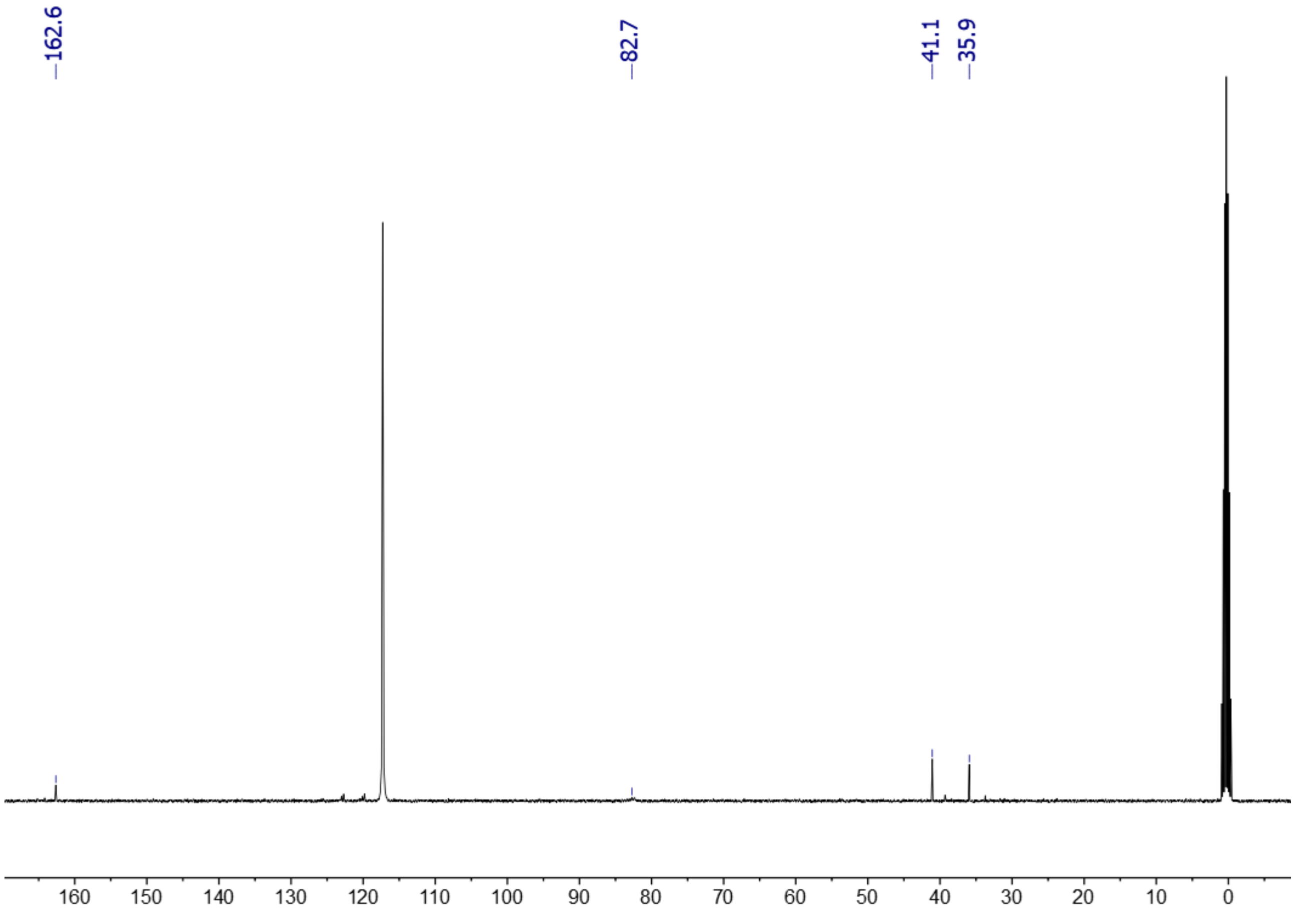

Fig. S2. $^{13}C\{^{1}H\}$ NMR spectrum (100.6 MHz) of crude **1**·Me_2NCHO in CD_3CN. Note the humps at ca. 120 ppm and at 82.7 ppm which turn into sharper signals in the ^{19}F decoupled spectrum below.

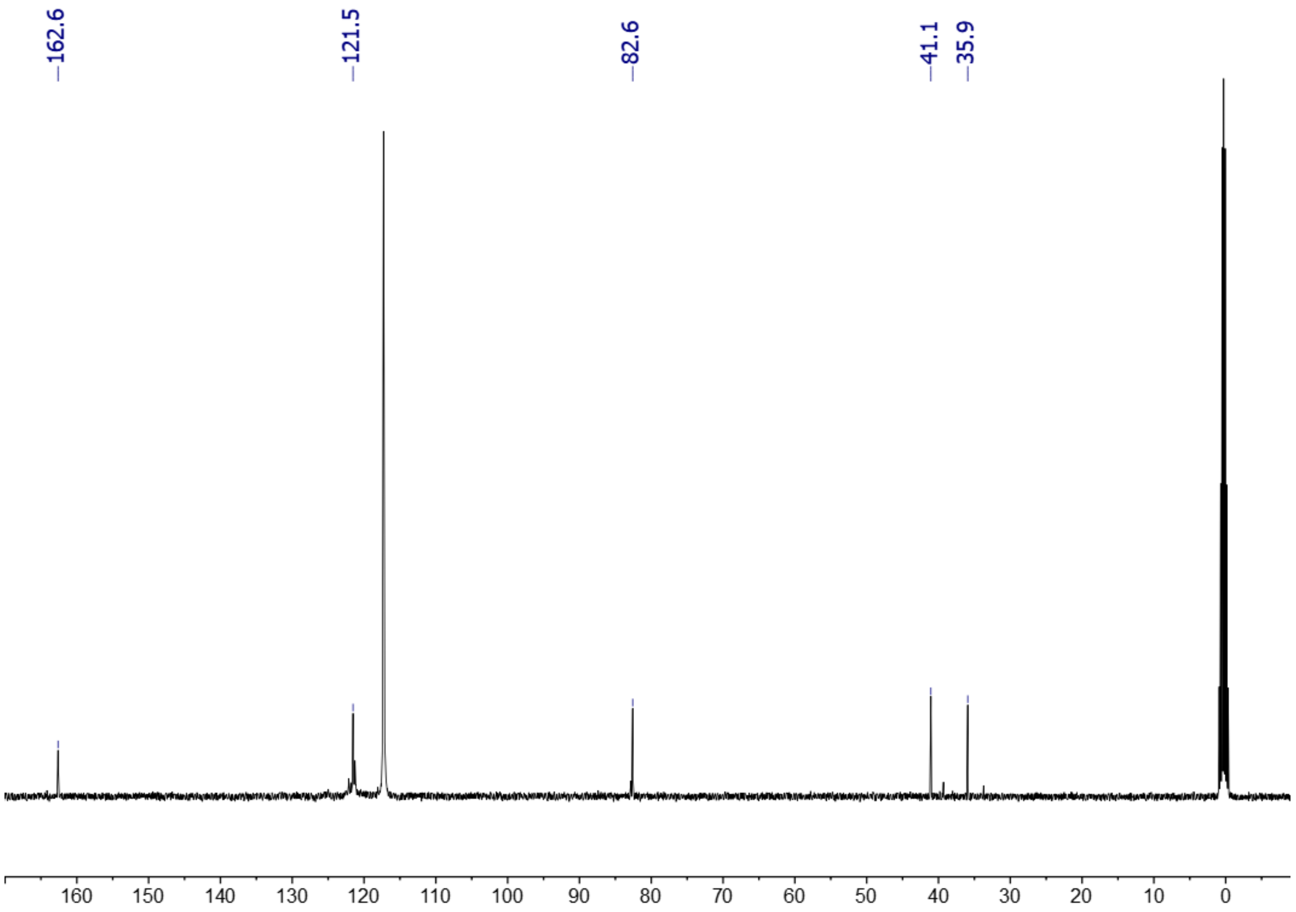

Fig. S3. $^{13}C\ \{^{1}H,^{19}F\}$ NMR spectrum (100.6 MHz) of crude **1**·Me_2NCHO in CD_3CN.

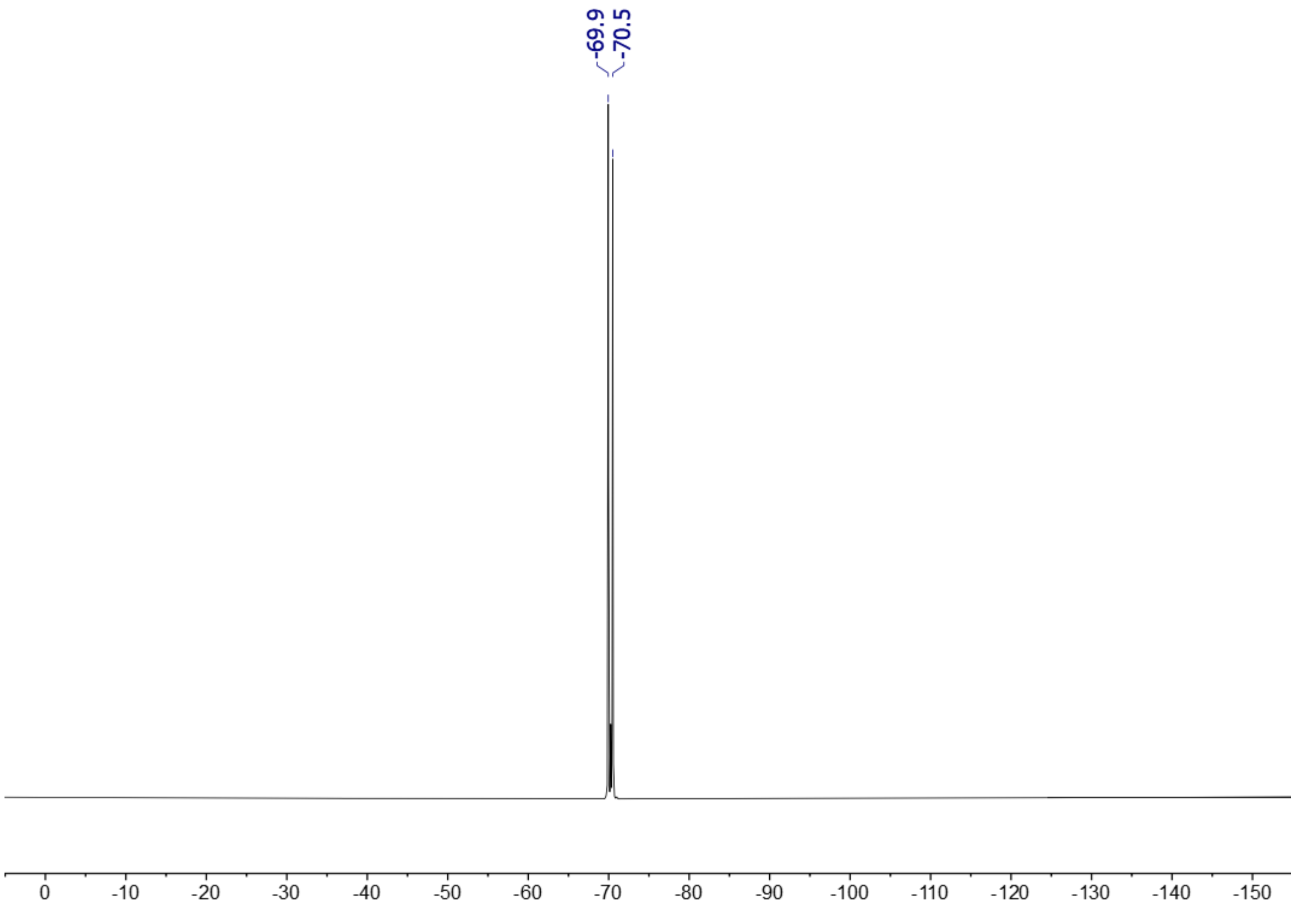

Fig. S4. ^{19}F NMR spectrum (100.6 MHz) of crude **1**·Me_2NCHO in CD_3CN.

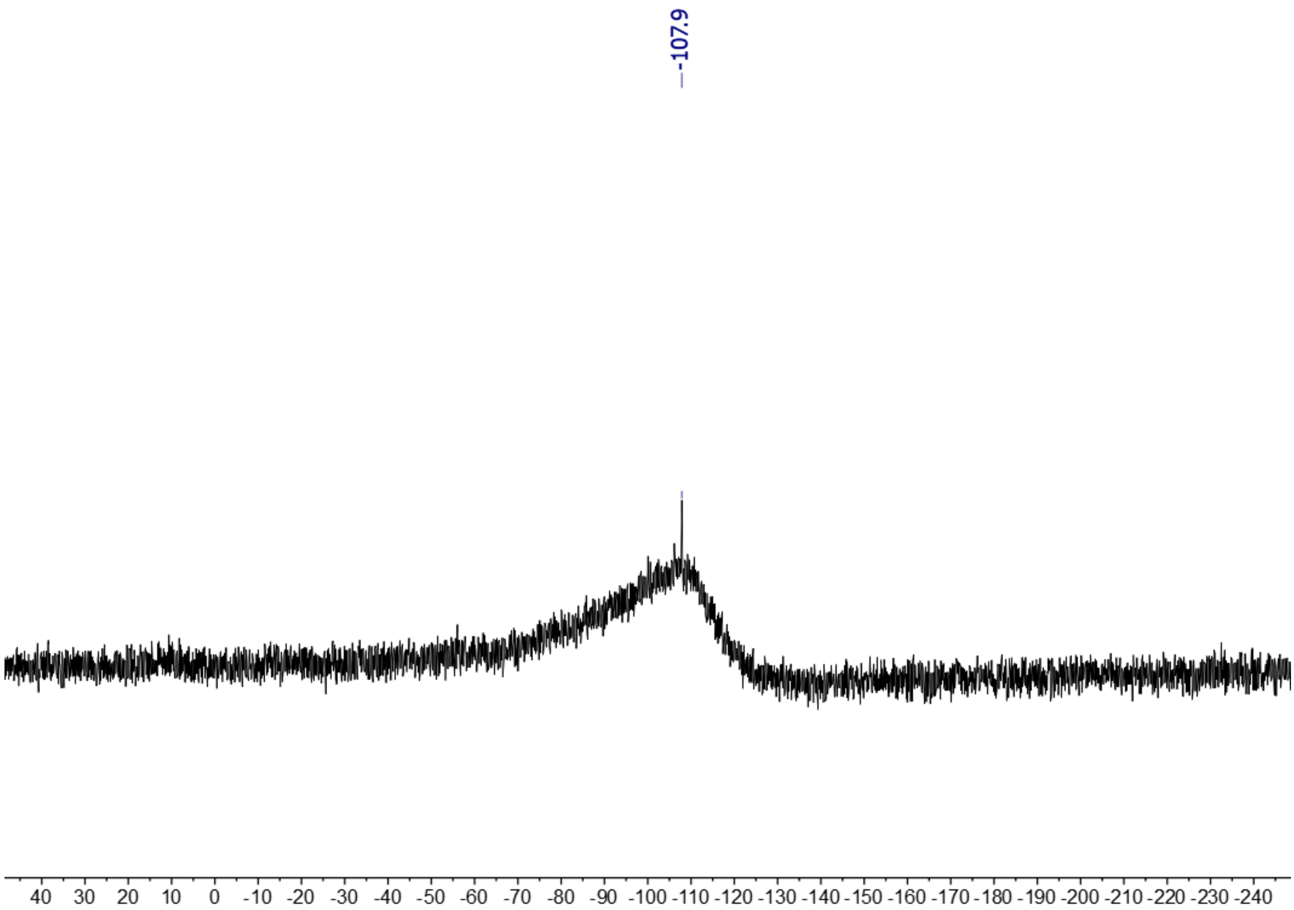

Fig. S5. $^{29}Si\{^1H\}$ NMR spectrum (79.5 MHz) of crude **1**·Me_2NCHO in CD_3CN.

2.2 $(pin^F)_2Ge\cdot Me_2SO$ ($2\cdot Me_2SO$)

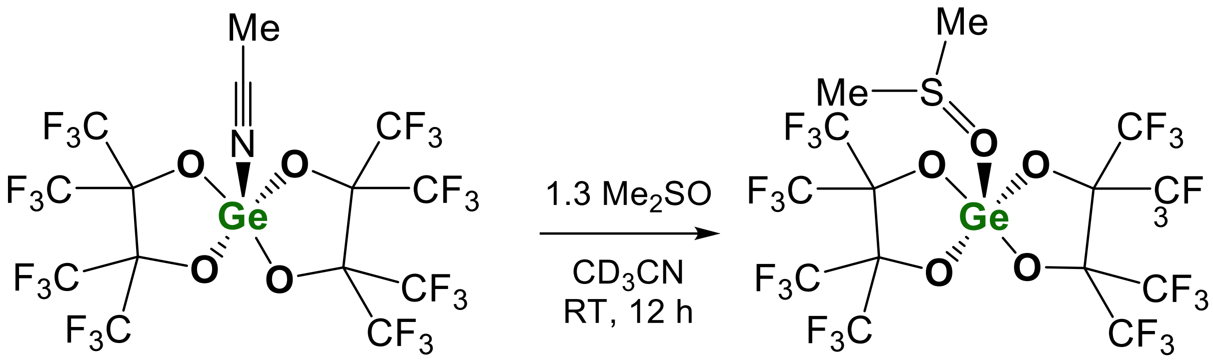

2·MeCN

2·Me_2SO
84 %

In the glovebox, a screwcap vial was charged with **2**·MeCN (0.193 g, 0.25 mmol, 1.0 eq.) and CD_3CN (0.660 g, 0.78 mL). DMSO (24.5 mg, 0.314 mmol, 1.3 eq) was added, the vial was closed and manually agitated (no stirrer bar included) until full dissolution of the solid (ca. 5 min). The reaction mixture was left for 12 h at room temperature followed by storage of the clear solution at −25 °C for 2 days. A crystalline chunk had formed and the cold mother liquor was withdrawn via syringe. After drying of the solid in vacuum 170 mg product (0.21 mmol, 84%) were transferred from the reaction vessel.

A similar procedure furnished single crystals suitable for X-ray structural study. The molecular structure is shown in Fig. S25.

^{1}H NMR (400.2 MHz, CD_3CN, 298 K): δ = 3.10 (s, 6 H, OSCH_3).
^{13}C{^{1}H} NMR (100.6 MHz, CD_3CN, 298 K): δ = 36.8 (OSCH$_3$).
^{13}C{^{1}H,^{19}F} NMR (100.6 MHz, CD_3CN, 298 K): δ = 122.6 (br, CF$_3$), 81.8 (OC(CF_3)$_2$), 36.8 (OSCH$_3$).
^{19}F NMR (376.6 MHz, CD_3CN, 298 K): δ = −69.76 (s, 12 F, CF_3), −70.31 (s, 12 F, CF_3).
CHNS elemental analysis calcd [%] for $C_{14}H_6F_{24}GeO_5S$ [814.85]**:** C 20.64, H 0.74, S 3.93; found [%]: C 21.25, H 0.39, S 3.84.

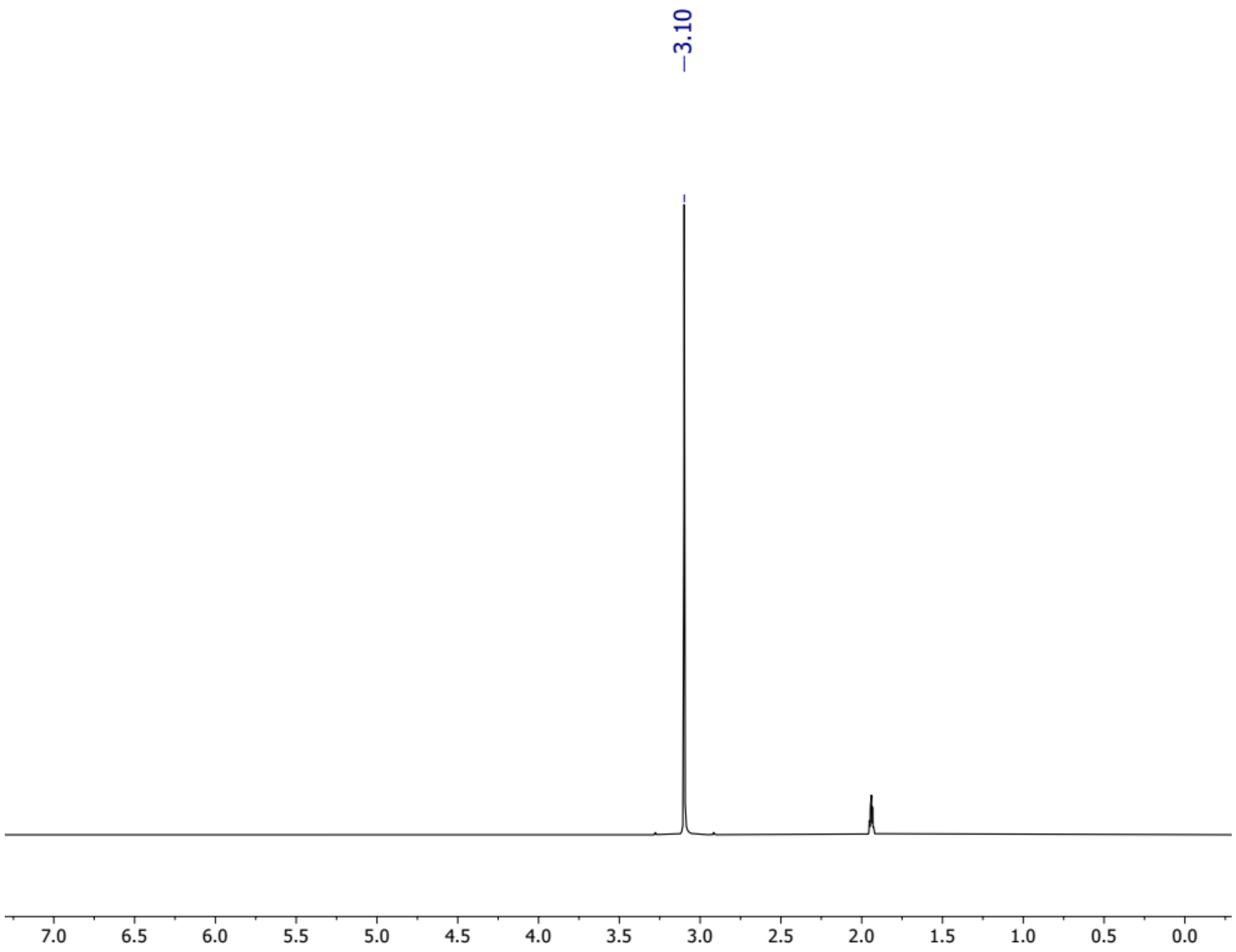

Fig. S6. ^{1}H NMR spectrum (400.2 MHz) of **2**·Me_2SO in CD_3CN. Note the $^1J_{CH}$ coupling of 144 Hz indicated by the ^{13}C satellite signal.

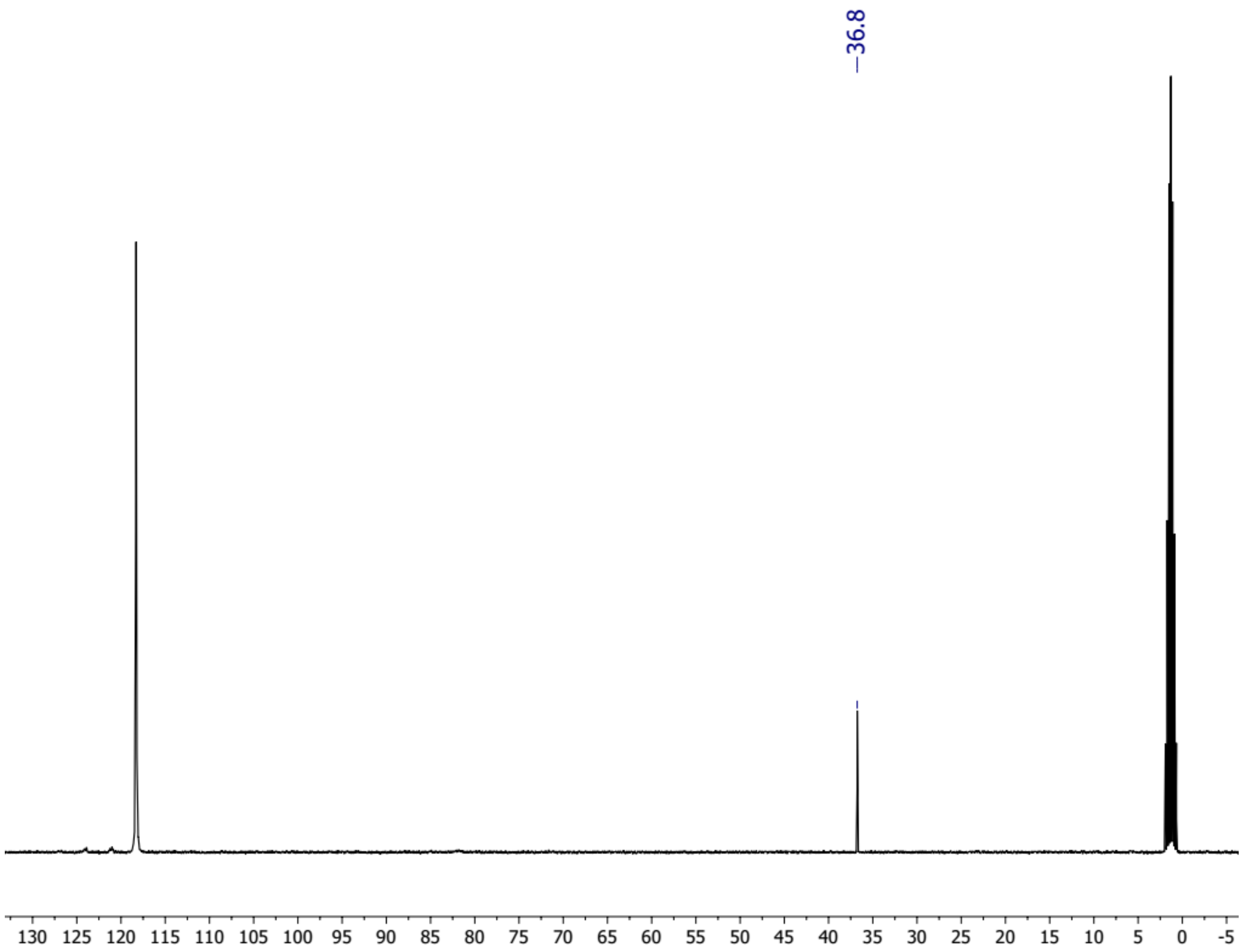

Fig. S7. $^{13}C\{^1H\}$ NMR spectrum (100.6 MHz) of **2**·Me_2SO in CD_3CN.

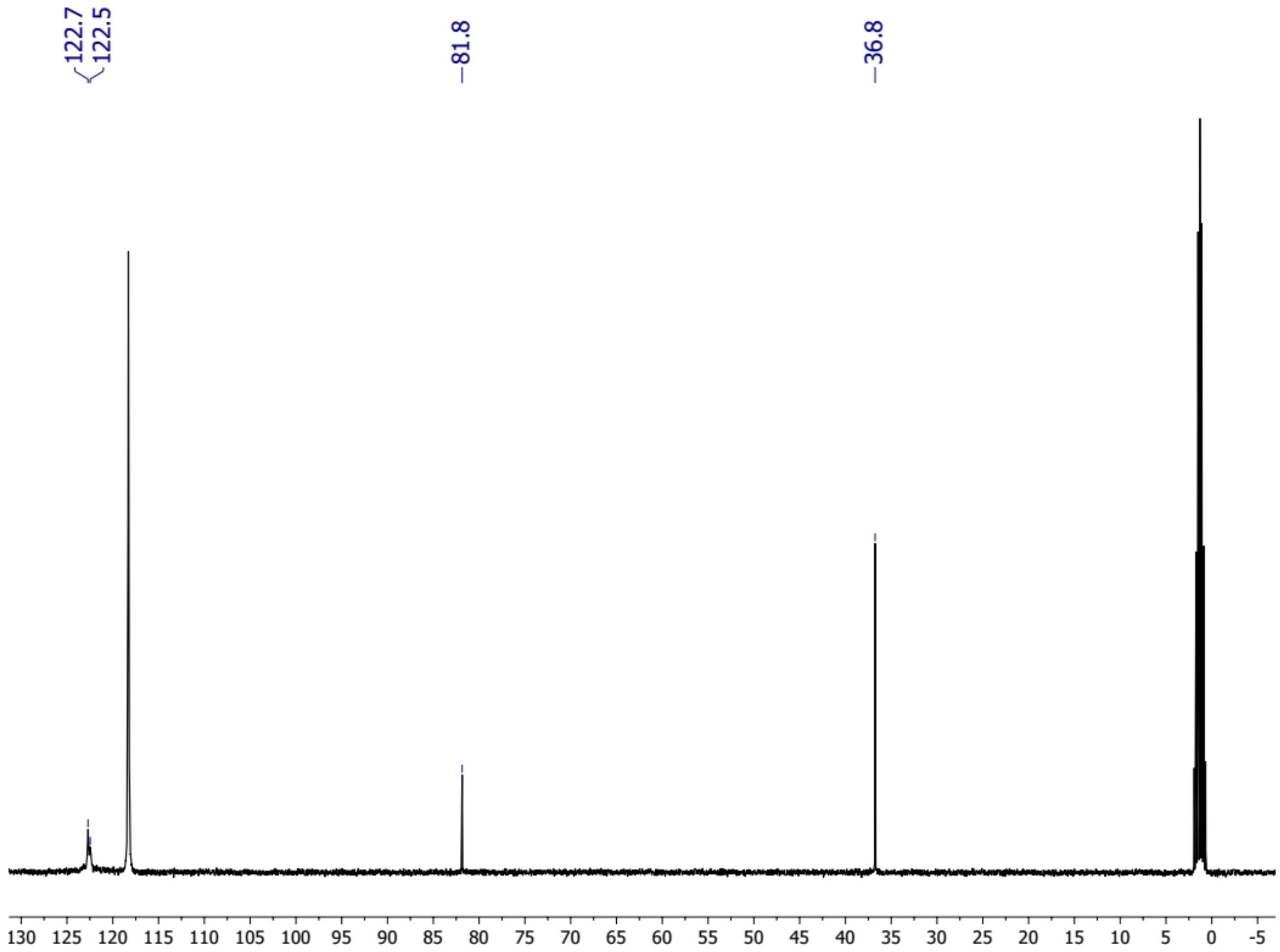

Fig. S8. $^{13}C\{^{1}H,^{19}F\}$ NMR spectrum (100.6 MHz) of **2**·Me_2SO in CD_3CN.

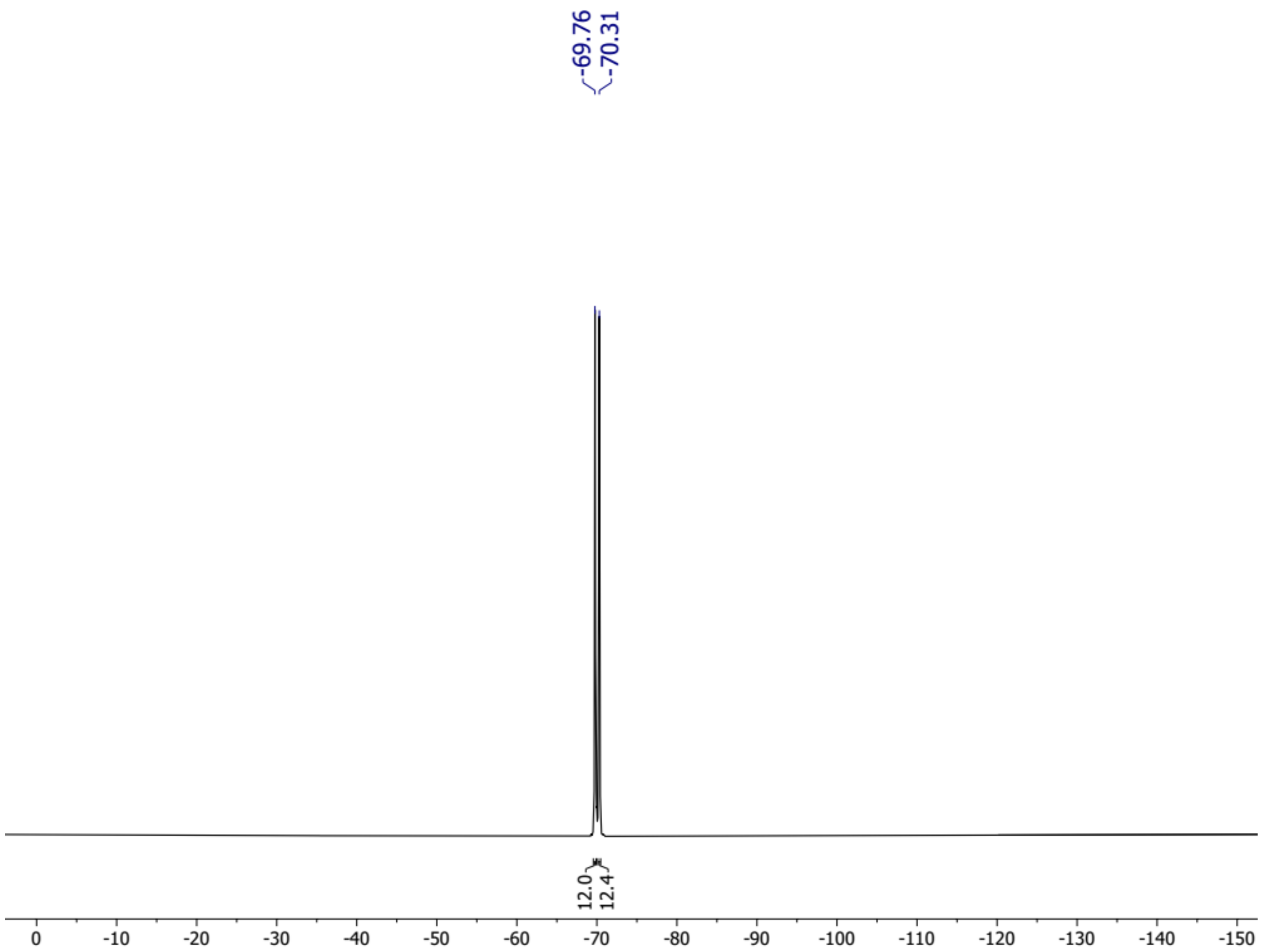

Fig. S9. ^{19}F NMR spectrum (376.6 MHz) of **2**·Me_2SO in CD_3CN.

Conversions of $(pin^F)_2Ge \cdot Me_2SO$ ($2 \cdot Me_2SO$) with DMF and Bu_3PO

To a solution of **2**·Me_2SO (38.7 mg, 47.5 µmol, 1.0 eq) in CD_3CN (0.5 mL) in a standard NMR sample tube was added DMF (6.8 mg, 93.0 µmol, 2.0 eq). The tube was closed with a rubber cap and mixed by manual agitation. After an induction period of ca. 1 h, proton NMR data was recorded that revealed a dynamic exchange between the donor molecules present in solution which was quick on the NMR timescale with only one signal set for each donor molecule and slight but notable shift of the ppm values with regard to the “free” donors (see Fig. S10). Re-recording the spectrum after several hours did not result in a notable change of the data. To the mixture was added Bu_3PO (11.1 mg, 50.8 µmol, 1.1 eq) and it was homogenized by manual agitation. After ca. 1 h the recorded NMR data (^1H, $^{31}P\{^1H\}$, see Fig. S11, S12) indicated major extrusion of the competing donors (*e.g.* CD_3CN, DMF, DMSO) from the Ge center by the phosphine oxide ($\delta(^{31}P)$ = 87.3 ppm for **2**·Bu_3PO, *cf.* $\delta(^{31}P)$ = 89.1 ppm for **2**·Et_3PO).[S15]

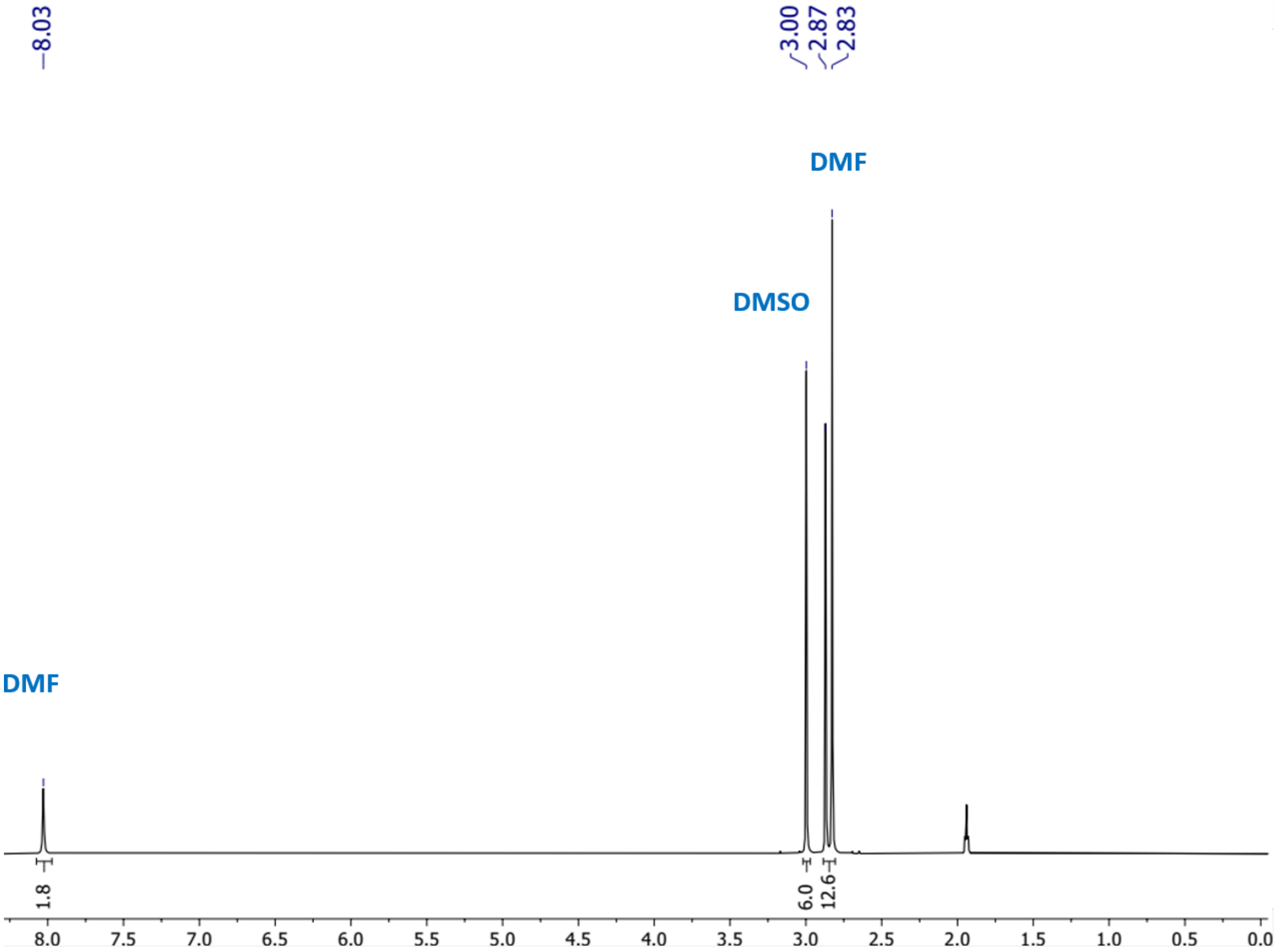

Fig. S10. ^1H NMR spectrum (400.2 MHz) of the mixture of **2**·Me_2SO and DMF (1.0 : 2.0 molar ratio) in CD_3CN.

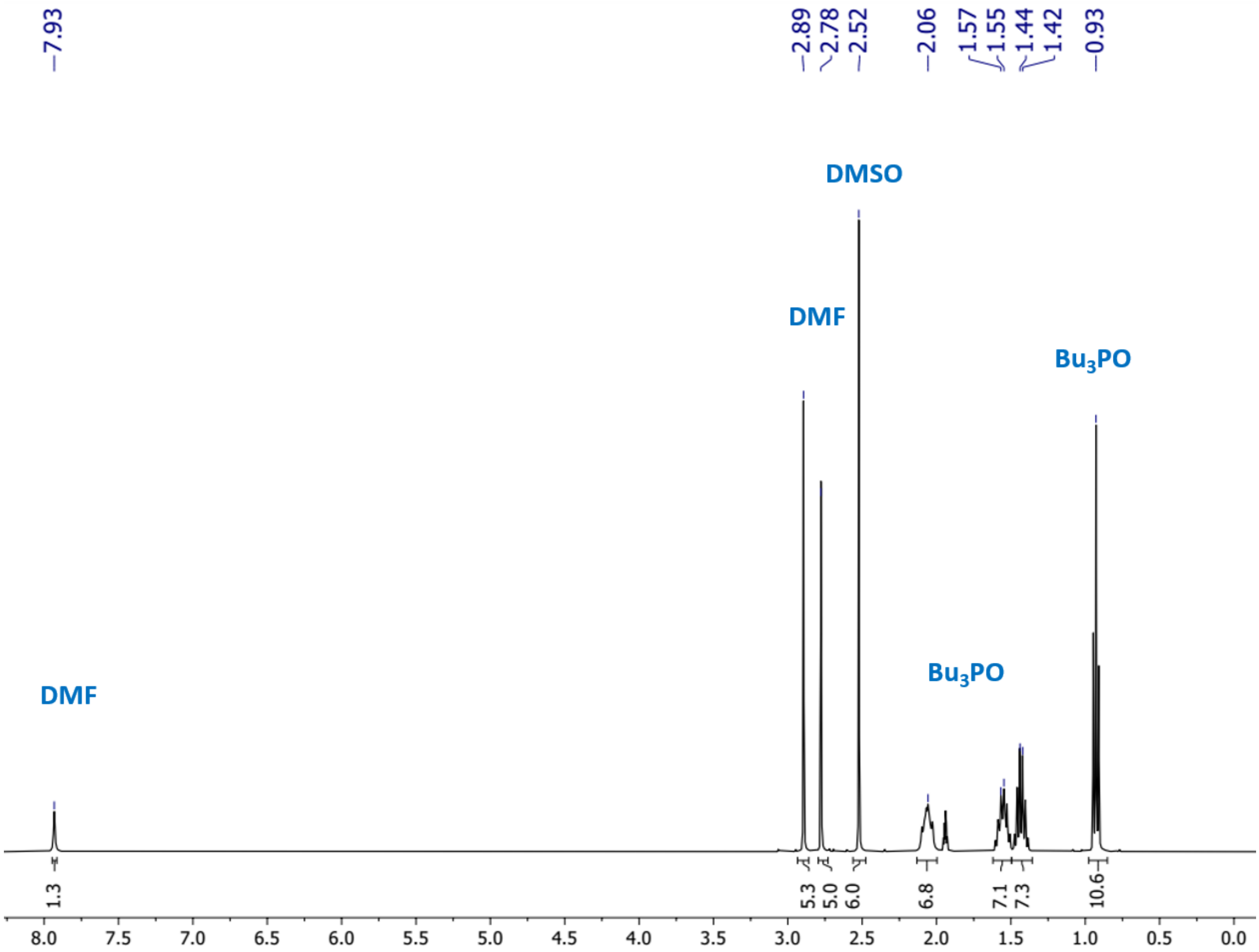

Fig. S11. ^{1}H NMR spectrum (400.2 MHz) of the mixture of **2**·Me_2SO, DMF and Bu_3PO (1.0 : 2.0 : 1.1 molar ratio) in CD_3CN.

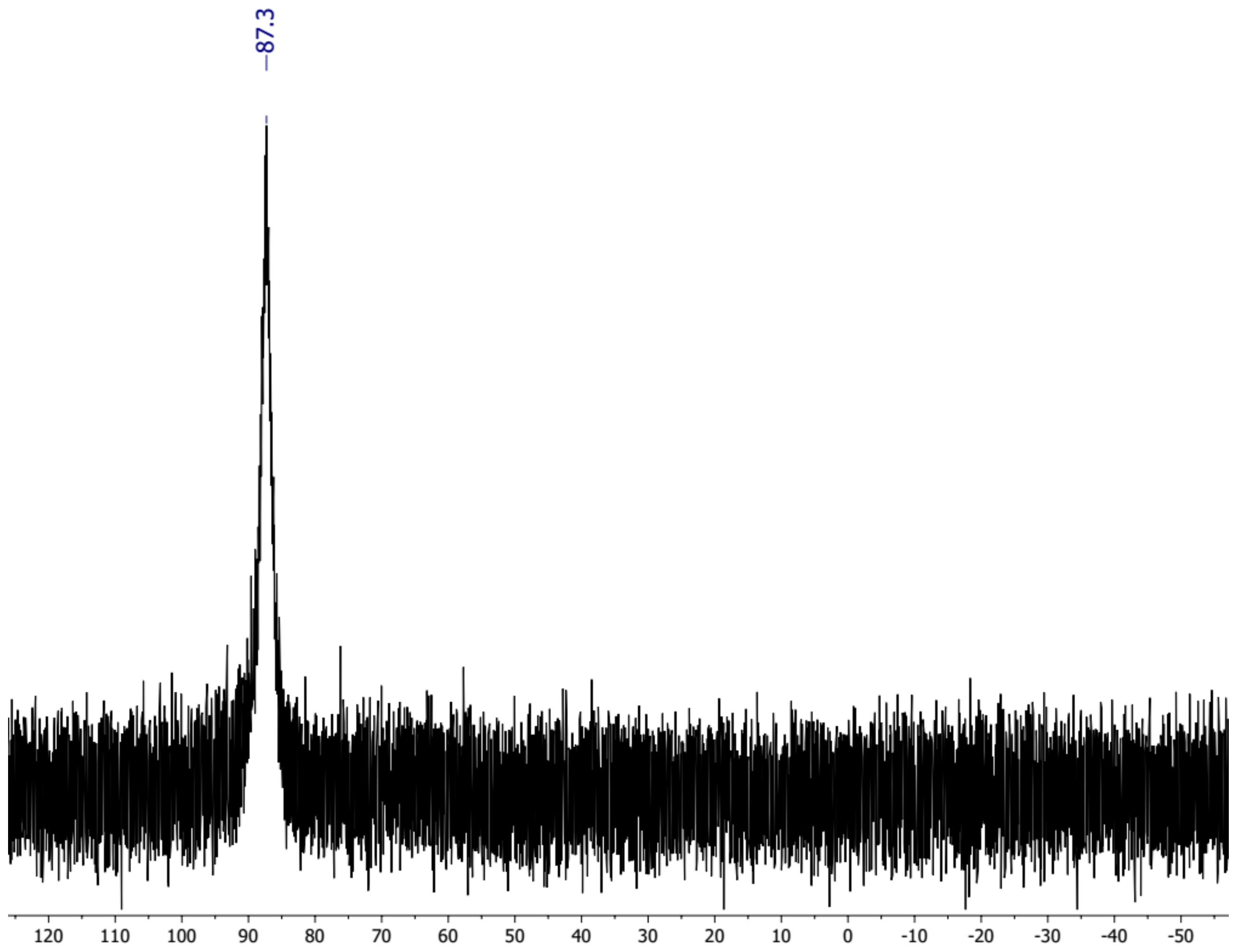

Fig. S12. $^{31}P\{^{1}H\}$ NMR spectrum (162.0 MHz) of the mixture of **2**·Me_2SO, DMF and Bu_3PO (1.0:2.0:1.1 molar ratio) in CD_3CN.

Conversions of $(pin^F)_2Ge\cdot Me_2SO$ ($2\cdot Me_2SO$) with $PhSiH_3$

An NMR sample tube was charged with **2**·Me_2SO (51.1 mg, 62.7 µmol) and CD_3CN (0.5 mL) and $PhSiH_3$ (10.2 mg, 94.3 µmol) was added to the mixture. The tube was closed with a rubber cap (supported with PTFE tape) and shaken for a minute to effect a homogenous solution. After recording of the initial NMR data the tube was placed in an oil-bath heated to 70 °C and the progress of the conversion was monitored in frequent intervals by NMR spectroscopy. Near-quantitative conversion of the DMSO group was found after 9 h at 70 °C.

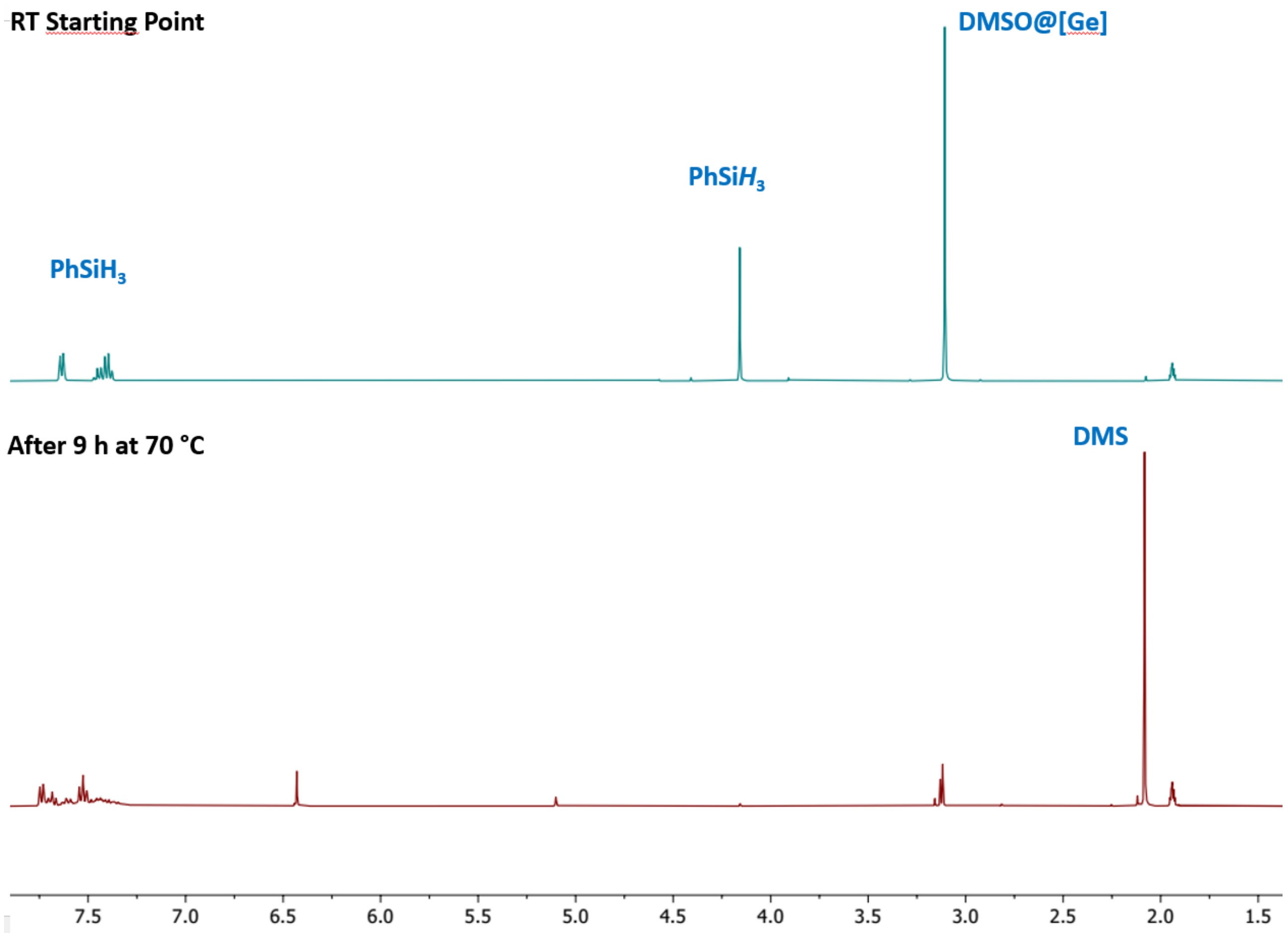

Fig. S13. Stacked ^1H NMR spectra (400.2 MHz) of the mixture of **2**·Me_2SO with $PhSiH_3$ (1.5 eq) in CD_3CN. Top: initial measurement after mixing at RT. Bottom: After heating the sample tube at 70 °C oil-bath temperature for 9 h.

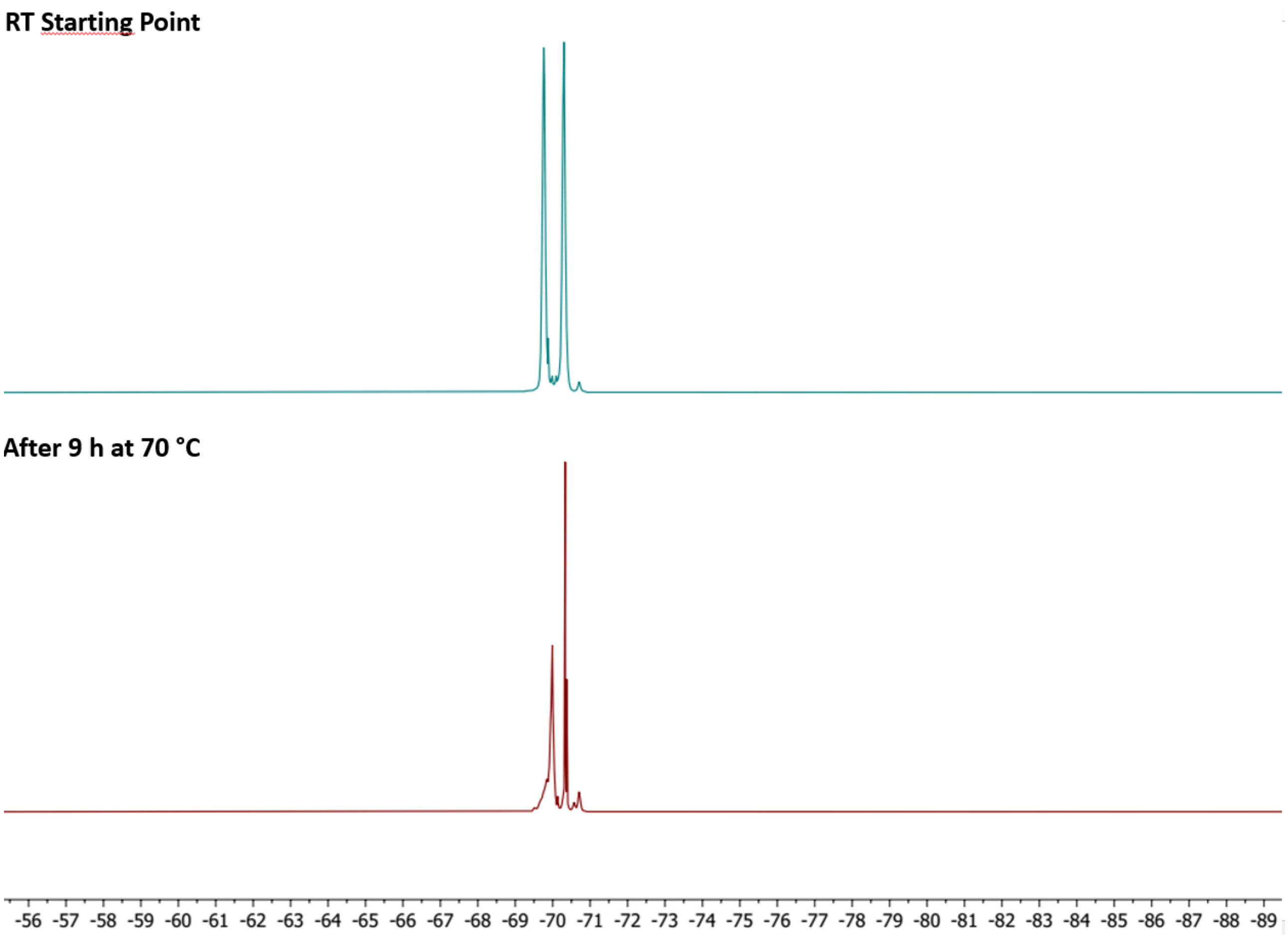

Fig. S14. Stacked ^{19}F NMR spectra (376.6 MHz) of the mixture of **2**·Me_2SO with $PhSiH_3$ (1.5 eq) in CD_3CN. Top: initial measurement after mixing at RT. Bottom: After heating the sample tube at 70 °C oil-bath temperature for 9 h. While the top spectrum shows the **2**·Me_2SO resonances the bottom spectrum indicates near-complete disintegration of the $(pin^F)_2Ge$ fragment.

2.3 Catalytic Reduction of Phosphine Oxides

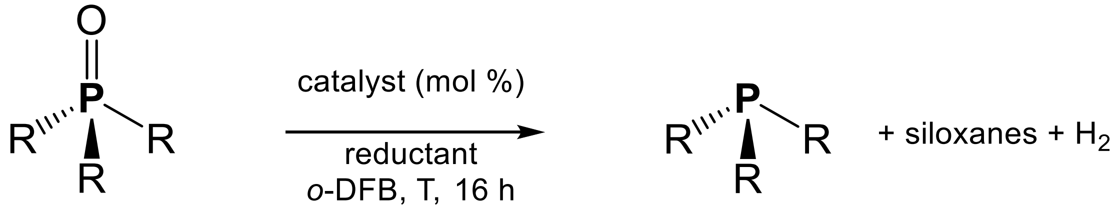

R = Me, Bu, Ph

catalyst = **1**·MeCN , **2**·MeCN, (**3**)$_2$·diox, $HNTf_2$
reductant = $PhSiH_3$, Et_3SiH, HBpin
T = 80 - 120 °C

General procedure for reduction catalysis reaction:

The catalyst and the phosphine oxide were dissolved in *o*-DFB. The reducing agent was added, and the reaction mixture was heated to the respective temperature for 16 hours. Tris(2,4-di-*tert*-butylphenyl) phosphite was added as an internal standard to determine yield via integration in the $^{31}P\{^1H\}$ NMR spectra. At this point, a sealed capillary filled with C_6D_6 was also included (to enable the automated NMR processing shim & lock routine of the sample). For the $^{31}P\{^1H\}$ NMR spectra used for quantification, a prolonged delay time between scans (5 s) and 100 scans were applied. Catalytic screening conditions are shown in Table S1. Example spectra of a monitored reaction is shown in Fig. S15.

Representative procedure for the catalytic reduction of R_3PO with $PhSiH_3$:

An NMR sample tube was charged with Bu_3PO (58.8 mg, 269.3 µmol, 1.0 eq) **1·**MeCN (9.9 mg, 14.0 µmol, 0.05 eq), *o*-DFB (0.5 mL) and the tube was shaken until all solid had dissolved. $PhSiH_3$ (47.8 mg, 441.7 µmol, 1.6 eq) was added under glovebox atmosphere. The sample was heated to 80 °C oil-bath temperature for a total of 16 h interrupted by few NMR data recordings for monitoring purpose. After the sample had cooled it was briefly reopened under glovebox atmosphere and tris(2,4-di-*tert*-butylphenyl) phosphite (17.5 mg, 27.1 µmol) was added as an internal standard. A 'lock capillary' filled with C_6D_6 was also added. The formation of Bu_3P was concluded from $^{31}P\{^1H\}$ NMR analysis. The consumption of Bu_3PO and formation of Bu_3P was quantified by comparing the (normalized) phosphorus nuclei ratios to the signals of the internal standard in the $^{31}P\{^1H\}$ NMR spectrum.

Table S1: Catalytic reduction of phosphine oxides to corresponding phosphines.

	R	Cat. (mol%)	Red (eq.)	T [°C]	Yield [%]
1	*n*Bu	**1**·MeCN (5)	$PhSiH_3$ (1.5)	80	97
2	*n*Bu	**2**·MeCN (5)	$PhSiH_3$ (1.5)	80	96
3	*n*Bu	**2**·MeCN (1)	$PhSiH_3$ (1.5)	80	75
4	*n*Bu	**2**·MeCN (5)	Et_3SiH (5.5)	110	< 1
5	*n*Bu	**2**·MeCN (5)	HBpin (4.5)	80	89
6	*n*Bu	(**3**)$_2$·diox (2.5)	$PhSiH_3$ (1.5)	80	52
7	*n*Bu	(**3**)$_2$·diox (2.5)	Et_3SiH (5)	120	< 1
8	*n*Bu	$HNTf_2$ (5)	$PhSiH_3$ (1.5)	80	99
9	*n*Bu	**None**	$PhSiH_3$ (1.5)	80	20
10	Me	**1**·MeCN (5)	$PhSiH_3$ (1.5)	80	91
11	Ph	**2**·MeCN (5)	$PhSiH_3$ (1.5)	80	69

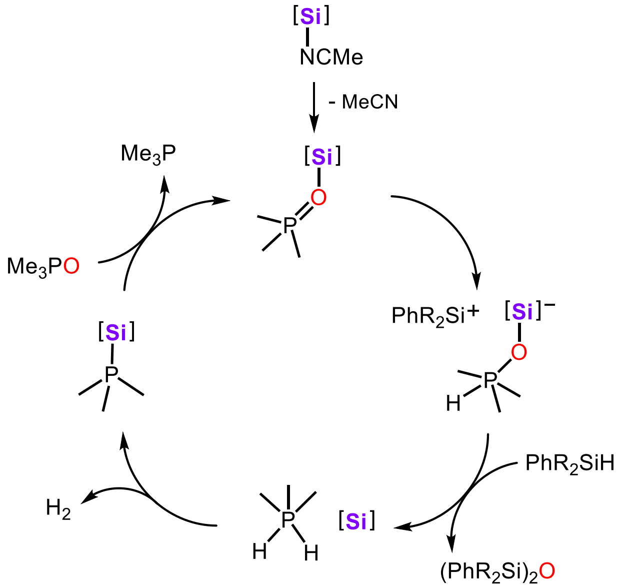

Scheme S1. Proposed mechanism for the Lewis acid catalyzed phosphine oxide reduction (R = H or $OSiPhR_2$). Similarly to account for the reduction of Me_2SO to Me_2S (Me_2NCHO may substantially differ).

Selected NMR spectra of Catalytic Phosphine Oxide Reduction

Conversion with **1**·MeCN Catalyst

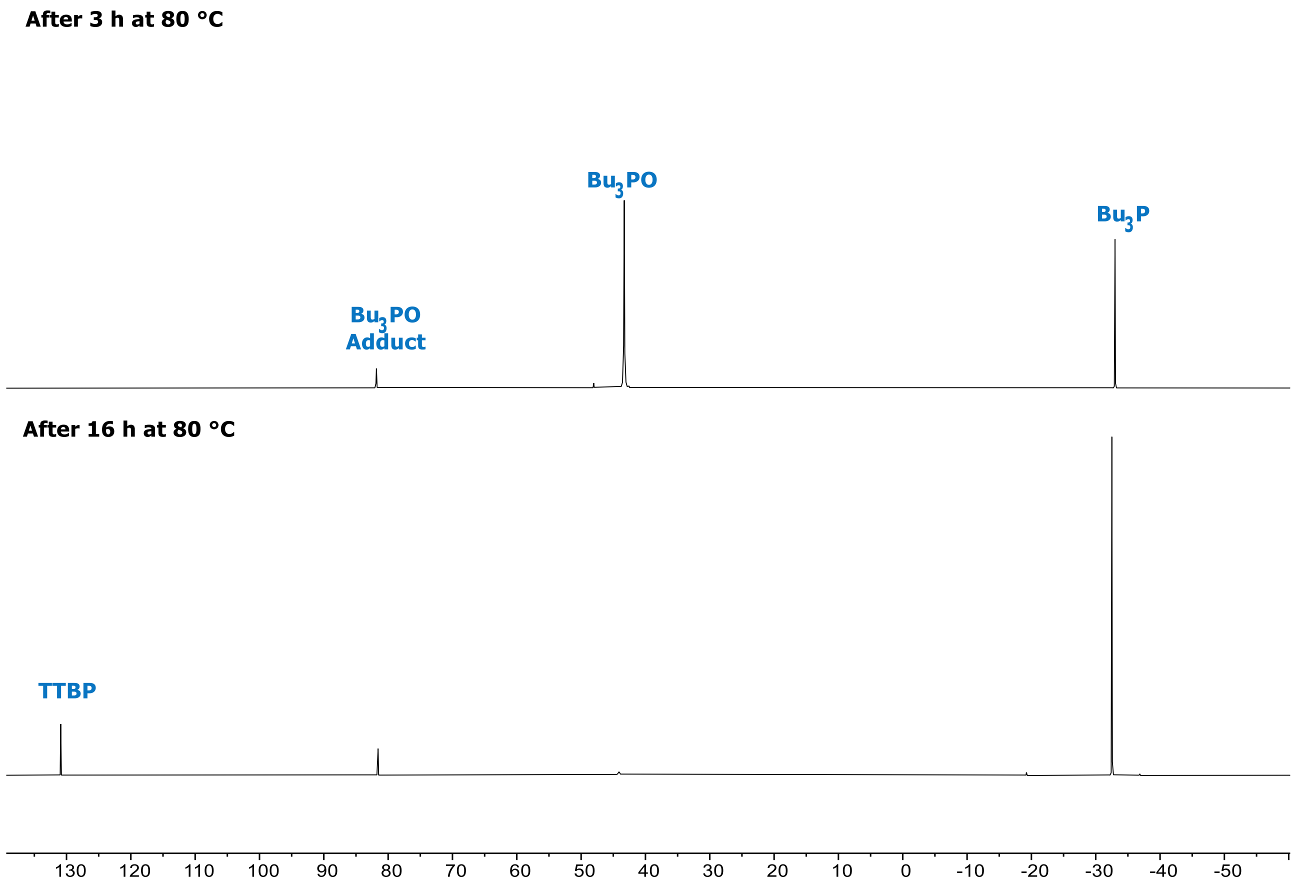

Fig. S15. Stacked $^{31}P\{^1H\}$ NMR spectra (162.0 MHz) of the catalytic reduction of Bu_3PO to Bu_3P described above in the representative procedure using **1**·MeCN (5 mol%), $PhSiH_3$ (1.5 eq) in *o*-DFB. TTBP = Tris(2,4-di-*tert*-butyl) phosphite. Top: Initial spectrum recorded after 3 h of heating to 80 °C. Bottom: End point spectrum recorded after 16 h at 80 °C reaction progress.

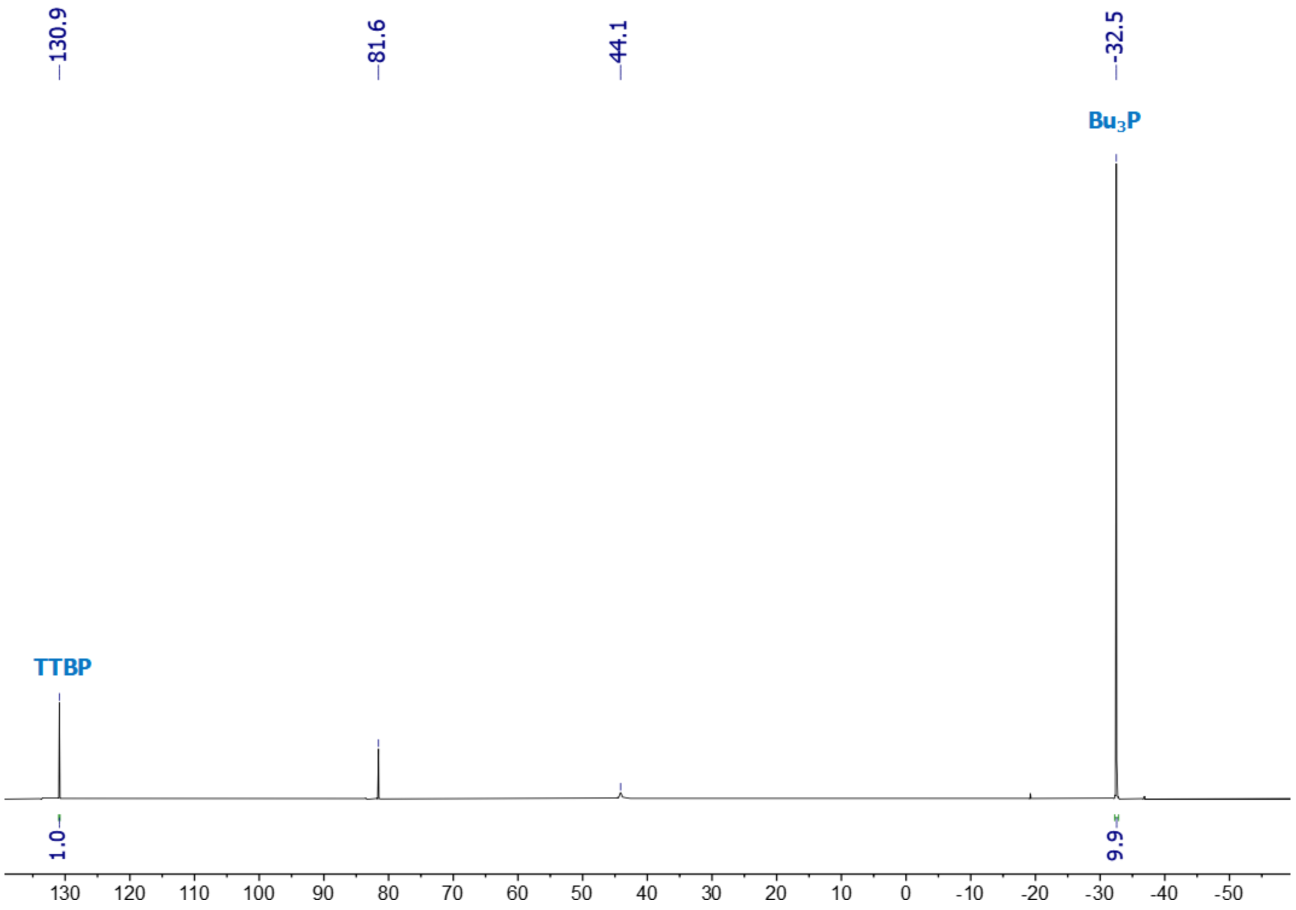

Fig. S16. $^{31}P\{^{1}H\}$ NMR spectrum (162.0 MHz) of the catalytic reduction of Bu_3PO to Bu_3P described above in the representative procedure using **1**·MeCN (5 mol%), $PhSiH_3$ (1.5 eq) in *o*-DFB. This is the bottom spectrum from the previous Figure S15 with Bu_3P and TTBP peaks picked and integrated for quantification purpose. TTBP = Tris(2,4-di-*tert*-butyl) phosphite.

2.4 Catalytic Reduction of DMSO

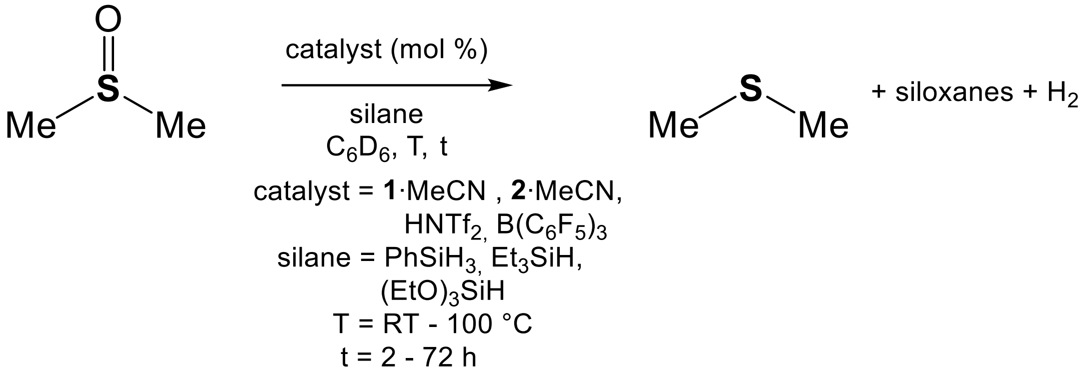

General procedure for the catalytic reduction of DMSO with silane:

An NMR sample tube was charged with the catalyst, the solvent[a], DMSO, and the silane[b] in this order. The tube was closed and shaken for few minutes until the catalyst had mostly dissolved. This was noted as the starting point of the reaction. The tube was charged with the internal standard 4,4'-di-*tert*-butyl-biphenyl (TBBP) and the mixture homogenized by manual agitation of the closed tube. Typical amounts can be concluded from the representative procedure below. For driving the reaction at elevated temperature the tube was dipped with a ca. 45° inclination into an oil bath at respective temperature with the upper filling level of the tube located ca. 1 cm below the surface of the oil. In cases of NMR monitoring over days with several data recordings the tube was shaken a couple of times between the measurements; otherwise the reactions were mostly diffusion controlled. In the ^{1}H NMR experiments used for quantification a prolonged delay time between scans (5 s) and 32 scans were applied. Catalytic screening outcomes are shown below in Table S2.

Notes: **a**: in cases of $PhSiH_3$ as reducing agent ca. 1/3 of the solvent was retained and used for washing the inner walls of the tubes after DMSO addition in order to avoid direct contact of neat $PhSiH_3$ and neat DMSO covering the surface. **b**: Use of $(EtO)_3SiH$ required a larger volume of silane to match the hydride equivalents of $PhSiH_3$ resulting in an overall higher dilution of the reaction mixture.

Representative procedure for the catalytic reduction of DMSO with silane:

An NMR sample tube was charged with **1**·MeCN (12.5 mg, 17.0 µmol, 0.03 eq), C_6D_6 (0.5 mL), Me_2SO (48.5 mg, 620.8 µmol, 1.0 eq), and $(EtO)_3SiH$ (336.1 mg, 2046.0 µmol, 3.3 eq). The tube was closed and shaken in frequent intervals over ca. 5 minutes until the catalyst had majorly dissolved. It was reopened briefly to introduce 4,4'-di-*tert*-butyl-biphenyl (22.3 mg, 83.7 µmol) and the internal standard was dissolved by shaking the tube for a minute. An initial ^{1}H NMR spectrum was recorded (which meant ca. 1 h room temperature exposition of the reaction) followed by heating the sample tube at 70 °C oil-bath temperature for a total of 18 h interrupted by few NMR data recordings for monitoring purpose. The formation of Me_2S (DMS) was concluded from ^{1}H and ^{13}C{^{1}H} NMR analysis. The consumption of DMSO and formation of DMS was quantified by comparing the (normalized) proton intensity ratios to the signals of the internal standard in the ^{1}H NMR spectrum. Near-quantitative consumption of DMSO was found of which 57% were converted to DMS (Table S2, Entry 14).

Table S2: Catalytic reduction of DMSO (dimethylsulfoxide) to DMS (dimethylsulfide) using hydrosilanes. This is a more detailed version of Table 2 from the main article. The latter had been polished with regard to the decimal digits in the reducing agent equivalents for comprehensive reasons. Table S2 includes the DMSO conversion ratio which in some instances markedly deviates from the DMS yield. The complemented information is in bold script. Cat. = catalyst, eq = equivalents, Rct. = Reaction, T = temperature, t = time.

	**Cat. (mol%)**	**Silane (eq)**	**Rct. T**	**Rct. t**	**Conversion[a] of DMSO [%]**	**Yield of[a] DMS [%]**
1	**1**·MeCN (5)	$PhSiH_3$ (**1.4**)	RT	2 h	**>99**	73
2	**1**·MeCN (3)	$PhSiH_3$ (**1.1**)	RT	4 h	**>99**	78
3	**1**·MeCN (1)	$PhSiH_3$ (1)	RT	4 h	NA	53[b]
4	**1**·MeCN (3)	$PhSiH_3$ (**0.7**)	RT	24 h	**85**	66[c]
5	**2**·MeCN (5)	$PhSiH_3$ (**1.5**)	RT	3 h	**19**	9
6	**2**·MeCN (3)	$PhSiH_3$ (**1.2**)	RT	4 h	**14**	6
7	**2**·DMSO (3)	$PhSiH_3$ (1)	RT	4 h	NA	12[d]
8	$B(C_6F_5)_3$ (5)	$PhSiH_3$ (**1.0**)	RT	18 h	**31**	13
9	$HNTf_2$ (5)	$PhSiH_3$ (**1.4**)	RT	2 h	**36**	12[e]
10	None	$PhSiH_3$ (**3.2**)	100 °C	24 h	**14**	8
11	**1**·MeCN (5)	Et_3SiH (**3.7**)	100 °C	24 h	**28**	6
12	**1**·MeCN (5)	$(EtO)_3SiH$ (**4.1**)	RT	72 h	**84**	52[f]
13	**1**·MeCN (5)	$(EtO)_3SiH$ (**3.2**)	RT	72h	**59**	40[g]
14	**1**·MeCN (3)	$(EtO)_3SiH$ (**3.3**)	70 °C	18 h	**>99**	57
15	**1**·MeCN (3)	$(EtO)_3SiH$ (**2.2**)	70 °C	36 h	**70**	39[h]
16	**1**·MeCN (1)	$(EtO)_3SiH$ (**4.4**)	RT	72 h	**39**	31
17	**2**·MeCN (5)	$(EtO)_3SiH$ (**3.9**)	RT	72 h	**26**	26
18	**2**·MeCN (3)	$(EtO)_3SiH$ (**3.3**)	70 °C	18 h	**24**	12
19	$HNTf_2$ (5)	$(EtO)_3SiH$ (**3.8**)	RT	72 h	**67**	10[e]
20	$HNTf_2$ (5)	$(EtO)_3SiH$ (**3.2**)	RT	72 h	**31**	11[i]
21	$B(C_6F_5)_3$ (5)	$(EtO)_3SiH$ (4)	RT	72 h	**9**	< 1
22	None	$(EtO)_3SiH$ (**5.2**)	100 °C	24 h	**<1**	< 1

a: Yield/conversion was determined by addition of 1,4-di-tert-butyl-biphenyl as internal standard. *b*: DMSO fully consumed and increased yield after 12 h. *c*: Monitoring for additional 6 h resulted only in negligible change. *d*: DMSO fully consumed and 78% yield after 13 h at 70 °C. *e*: An oil separated from the mixture. *f*: After a total of 96 h DMSO was fully consumed and 58% yield was found. *g*: $CDCl_3$ was used as solvent instead of C_6D_6. *h*: No further conversion by additional heating for 12 h. *i*: $CDCl_3$ was used as solvent and a solid separated after few hours.

Selected NMR spectra of Catalytic DMSO Reductions.

<u>Conversion with **1**·MeCN Catalyst and $(EtO)_3SiH$ Reducing Agent.</u>

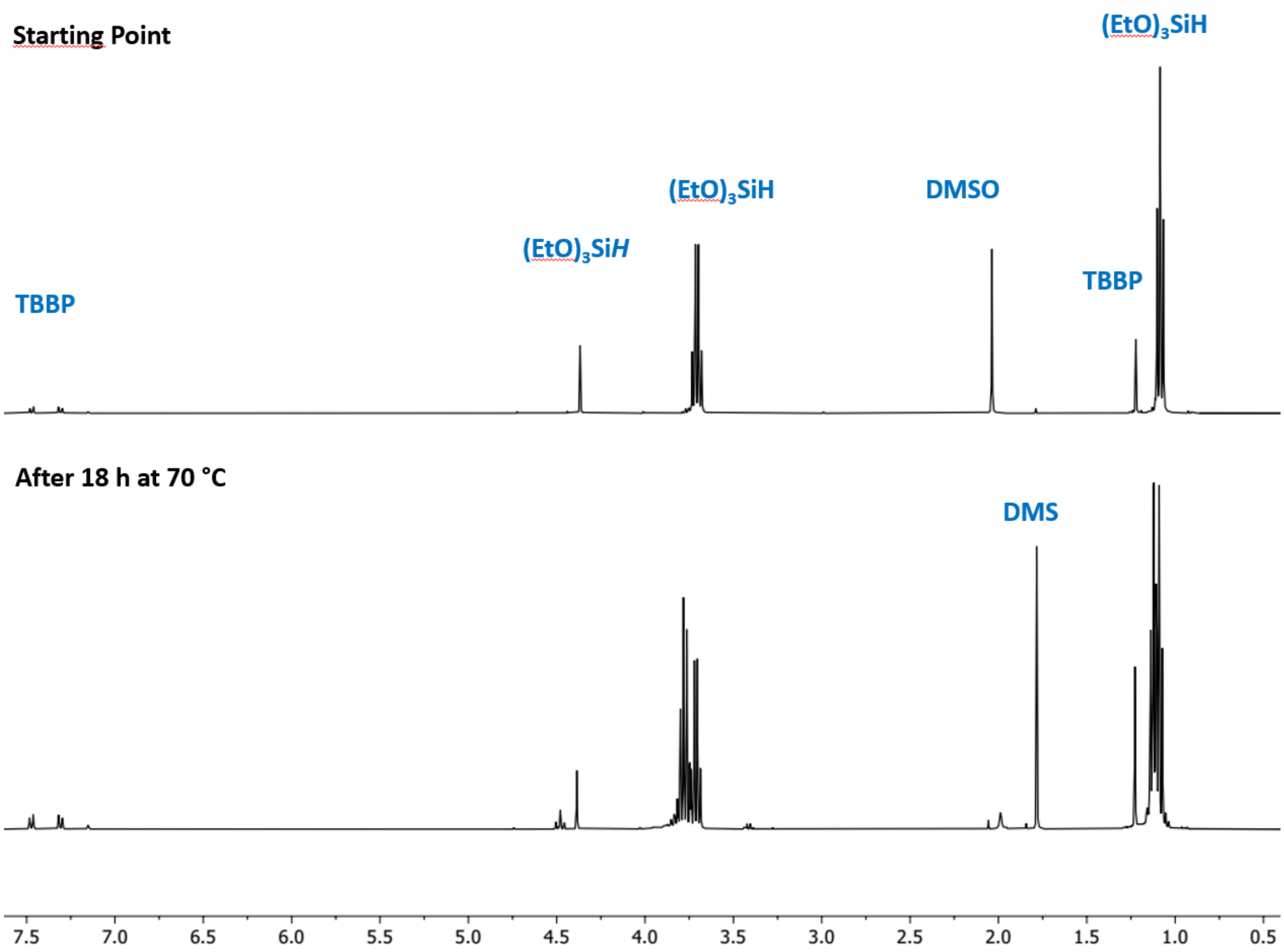

Fig. S17. Stacked ^1H NMR spectra (400.2 MHz) of the catalytic reduction of DMSO to DMS described above in the representative procedure using **1**·MeCN (3 mol%), $(EtO)_3SiH$ (3 eq) in C_6D_6 (0.5 mL). TBBP = 4,4'-di-*tert*-butyl-biphenyl. Top: Initial spectrum recorded ca. 1 h after mixing. Bottom: End point spectrum recorded after 18 h at 70 °C reaction progress.

Note: the chemical shift of the signals is not referenced and, in addition, is not expected to strictly match the literature values of, for example, DMSO in C_6D_6 because the larger volume of the silane attributes C_6D_6/silane mixed-medium characteristics to the sample.

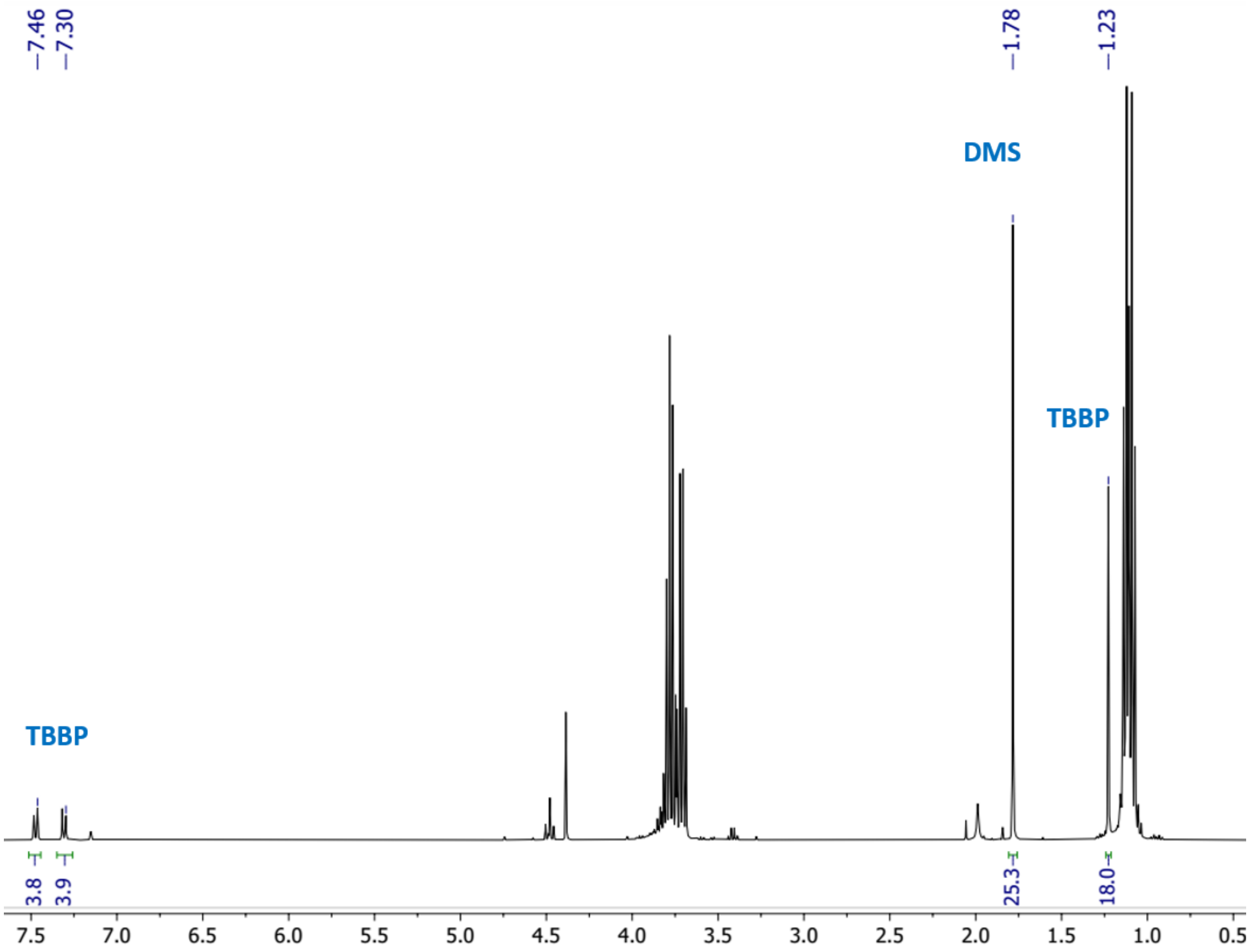

Fig. S18. ^{1}H NMR spectrum (400.2 MHz) of the catalytic reduction of DMSO to DMS described above in the representative procedure using **1**·MeCN (3 mol%) and $(EtO)_3SiH$ (3 eq) in C_6D_6. This is the bottom spectrum from the previous Figure S17 with DMS and TBBP peaks picked and integrated for quantification purpose.

Conversion with $B(C_6F_5)_3$ Catalyst and $PhSiH_3$ Reducing Agent

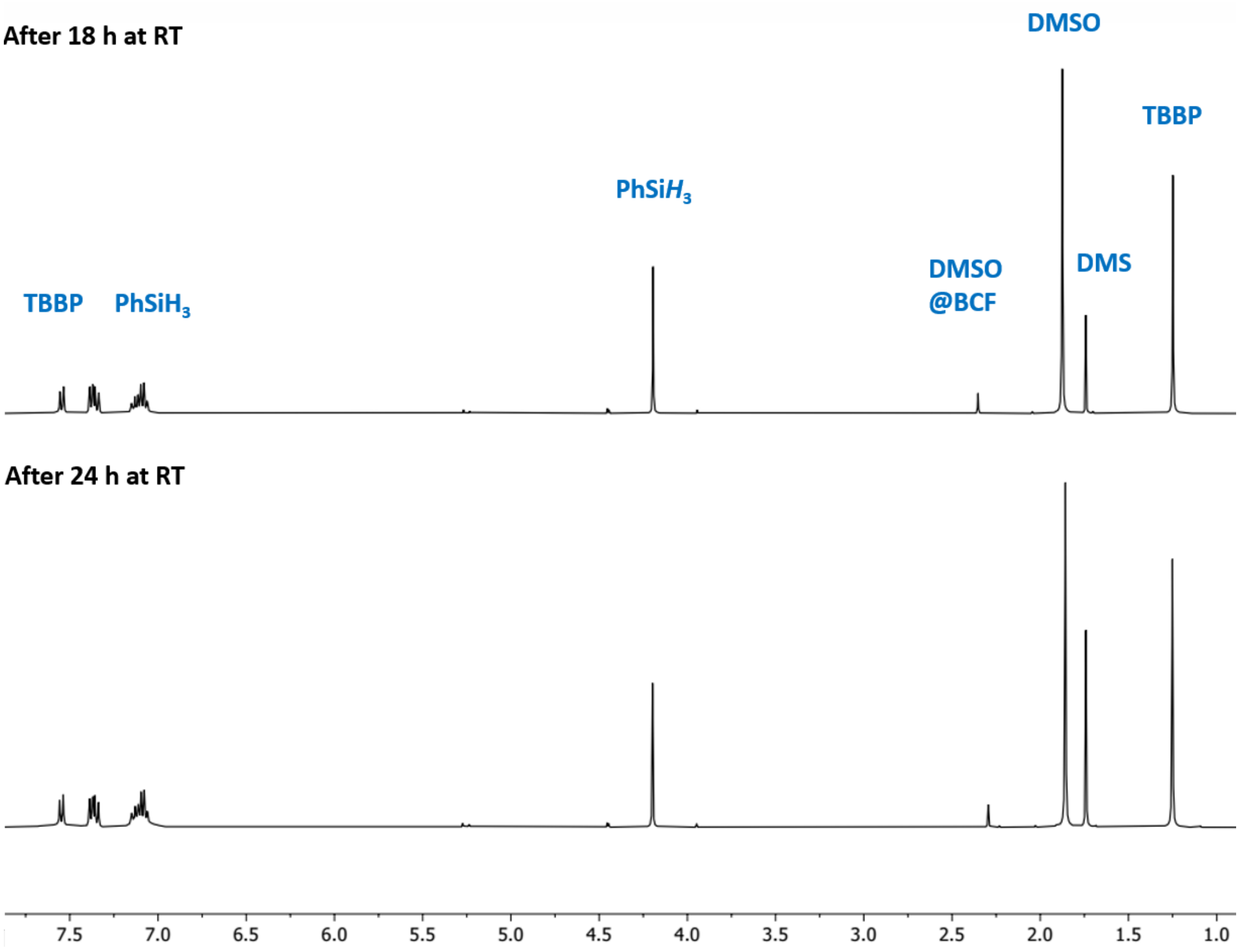

Fig. S19. Stacked ^{1}H NMR spectra (400.2 MHz) of the catalytic reduction of DMSO to DMS using $B(C_6F_5)_3$ (BCF, 5 mol%), $PhSiH_3$ (1 eq) in C_6D_6. TBBP = 4,4'-di-*tert*-butyl-biphenyl. Top: After 18 h reaction progress at RT. Bottom: After 24 h reaction progress at RT.

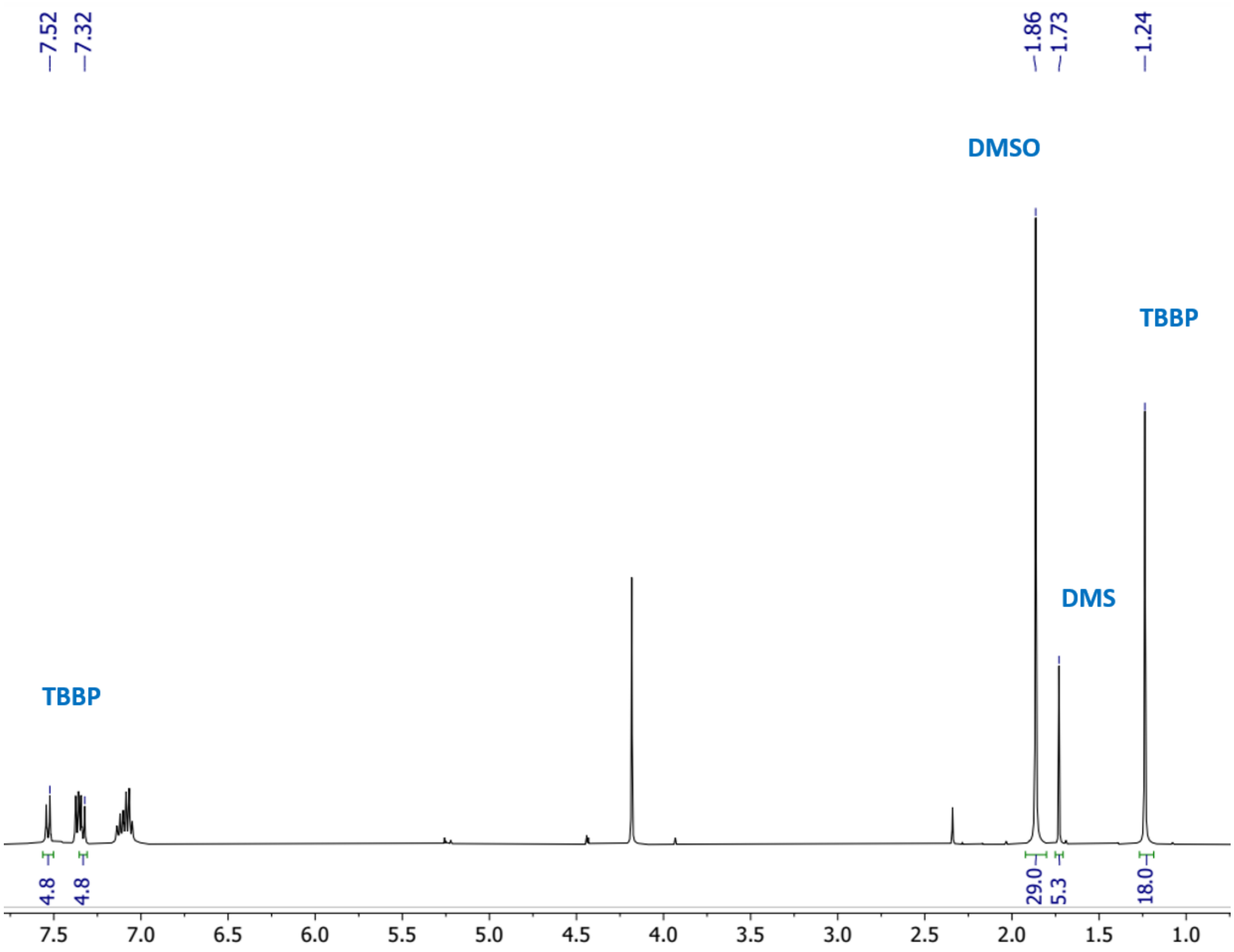

Fig. S20. ^{1}H NMR spectrum (400.2 MHz) of the catalytic reduction of DMSO to DMS using BCF (5 mol%) and $PhSiH_3$ (1 eq) in C_6D_6. This is the top spectrum from the previous Figure S19 with DMSO, DMS and TBBP peaks picked and integrated for quantification purpose. The spectrum is referenced to the chemical shift of DMS.

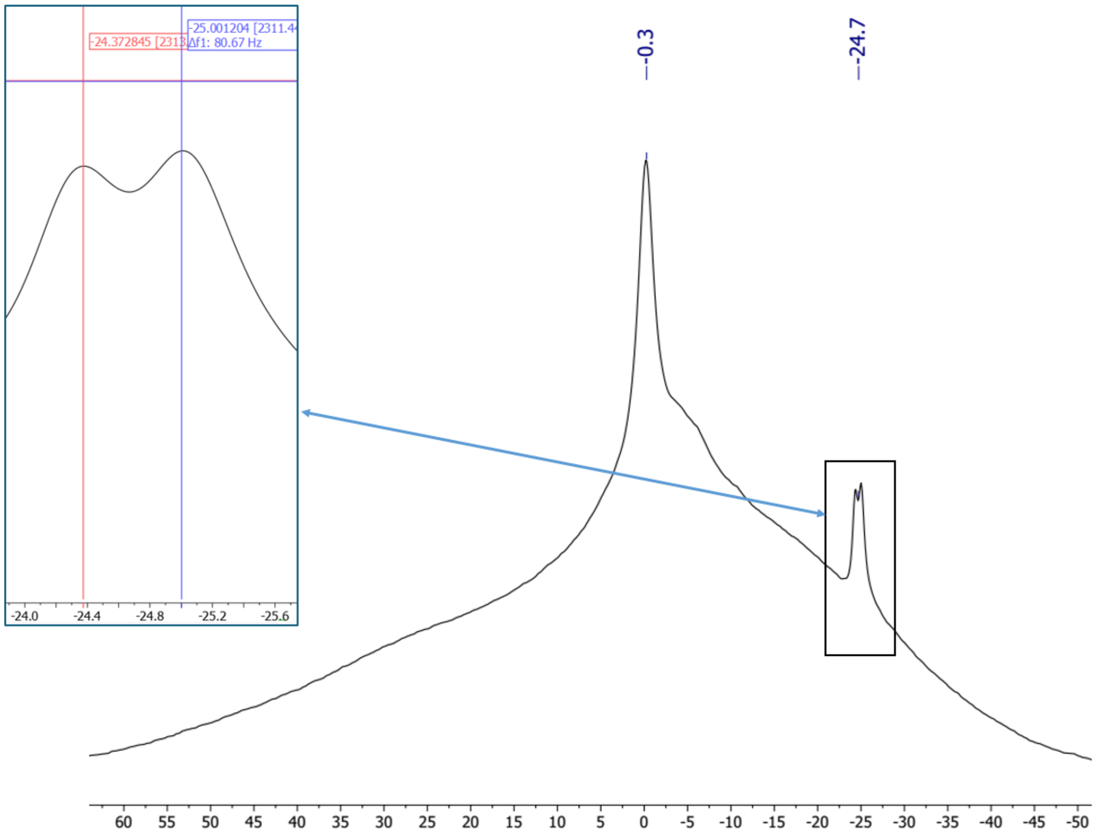

Fig. S21. ^{11}B NMR spectrum (128.4 MHz) of the catalytic reduction of DMSO to DMS using BCF (5 mol%) and $PhSiH_3$ (1 eq) in C_6D_6. The data was recorded after 18 h at RT reaction progress. The dublet at −24.7 ppm is assigned to $[HB(C_6F_5)_3]^-$ with a $^1J_{BH}$ coupling of 81 Hz. The signal at −0.3 ppm is assumed to originate from uncharged Donor·$B(C_6F_5)_3$ species (Donor = MeCN, DMSO, DMS, etc.)

2.5 Catalytic Reduction of DMF

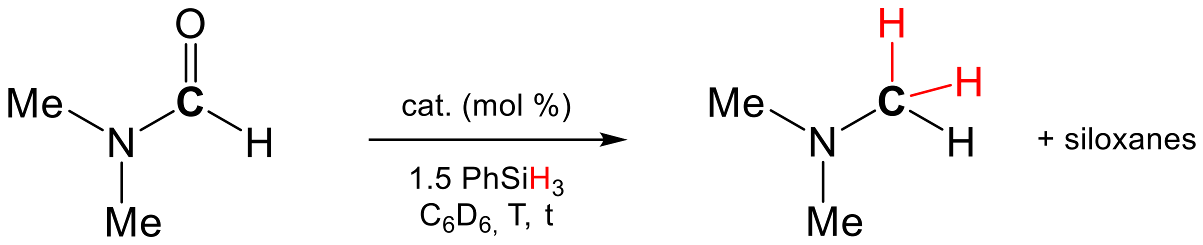

catalyst = **1**·MeCN , **2**·MeCN, $B(C_6F_5)_3$
T = 80 - 110 °C
t = 12 - 48 h

General procedure for reduction catalysis reaction:

Catalyst, DMF (0.03 mmol, 1.0 eq.), and a known amount of 4,4'-di-*tert*-butyl-biphenyl were dissolved in C_6D_6 (0.5 mL). $PhSiH_3$ (1.5 eq.) was added, the reaction mixture was heated to the desired temperature and monitored via ^1H NMR spectroscopy. All catalytic screening conditions are shown in Table S3. An example spectrum of a monitored reaction is shown below (Fig. S22, S23).

Representative procedure for the catalytic reduction of DMF with $PhSiH_3$:

An NMR sample tube was charged with **1**·MeCN (9.1 mg, 12.4 μmol, 0.05 eq), C_6D_6 (0.5 mL), Me_2NCHO (18 mg, 246 μmol, 1.0 eq), and $PhSiH_3$ (40.7 mg, 376.1 μmol, 1.5 eq). The tube was closed and shaken until the catalyst had fully dissolved. It was reopened briefly to introduce 4,4'-di-*tert*-butyl-biphenyl (16.4 mg, 61.6 μmol) and the internal standard dissolved by shaking the tube for a minute. An initial ^1H NMR spectrum was recorded (which meant ca. 1 h room temperature exposition of the reaction) followed by heating the sample tube at 80 °C oil-bath temperature for a total of 24 h interrupted by few NMR data recordings for monitoring purpose. The formation of Me_3N was concluded from ^1H analysis. The consumption of DMF and formation of Me_3N was quantified by comparing the (normalized) proton intensity ratios to the signals of the internal standard in the ^1H NMR spectrum.

Table S3: Catalytic reduction of DMF to Me_3N using $PhSiH_3$.

	**Cat. (mol%)**	**Solvent**	**T [°C]**	**t**	**Yield [%]**
1	**1**·MeCN (5)	C_7D_8	110	12 h	99
2	**1**·MeCN (5)	C_6D_6	80	24 h	71
3	**2**·MeCN (5)	C_6D_6	80	24 h	61
4	$B(C_6F_5)_3$ (5)	C_6D_6	80	22 h	70
5	**None**	*o*-DFB	110	48 h	0

Selected NMR spectra of Catalytic DMF Reduction

Conversion with **1**·MeCN Catalyst

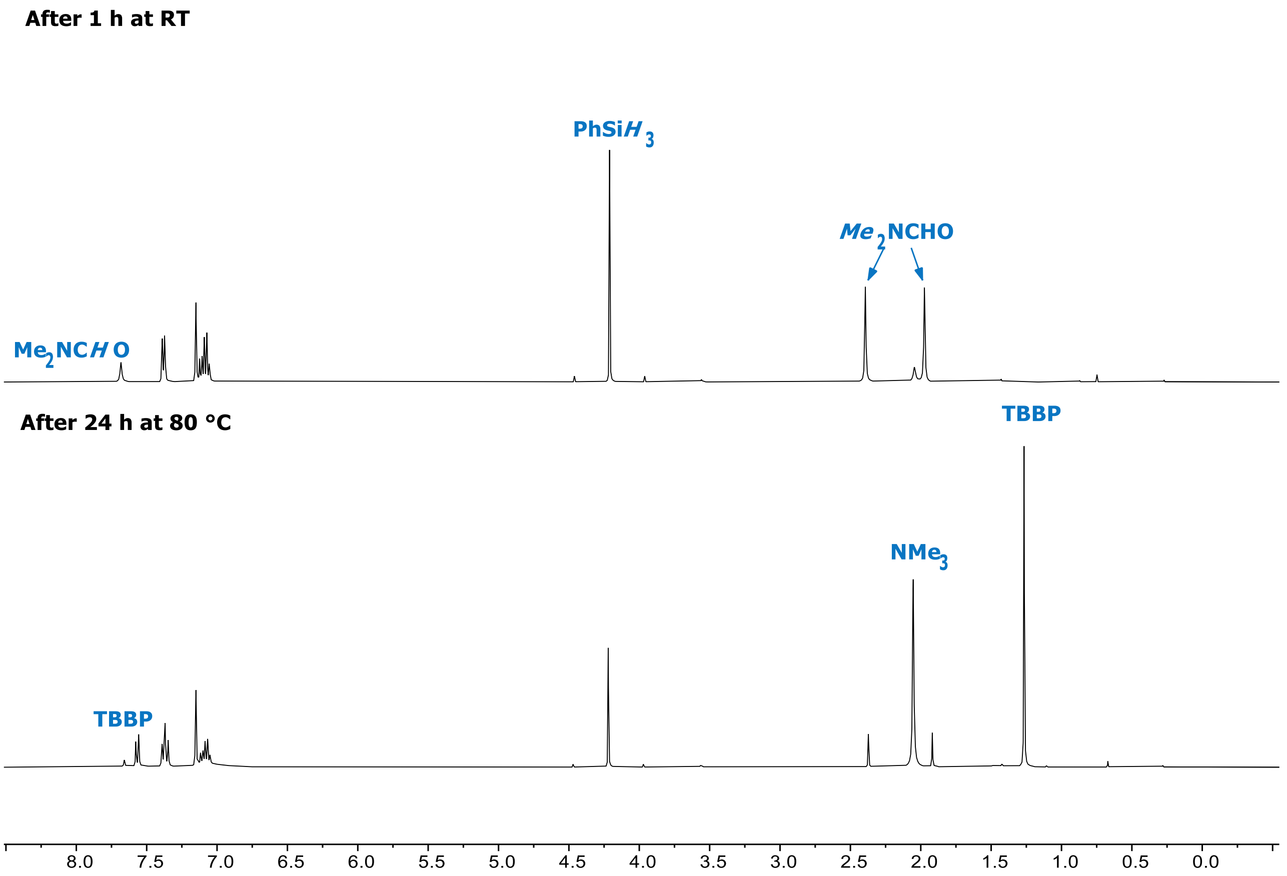

Fig. S22. Stacked ^{1}H NMR spectra (400.2 MHz) of the catalytic reduction of DMF to Me_3N described above in the representative procedure using **1**·MeCN (5 mol%), $PhSiH_3$ (1.5 eq) in C_6D_6. TBBP = 4,4'-di-*tert*-butyl-biphenyl. Top: Initial spectrum recorded ca. 1 h after mixing. Bottom: End point spectrum recorded after 24 h at 80 °C reaction progress.

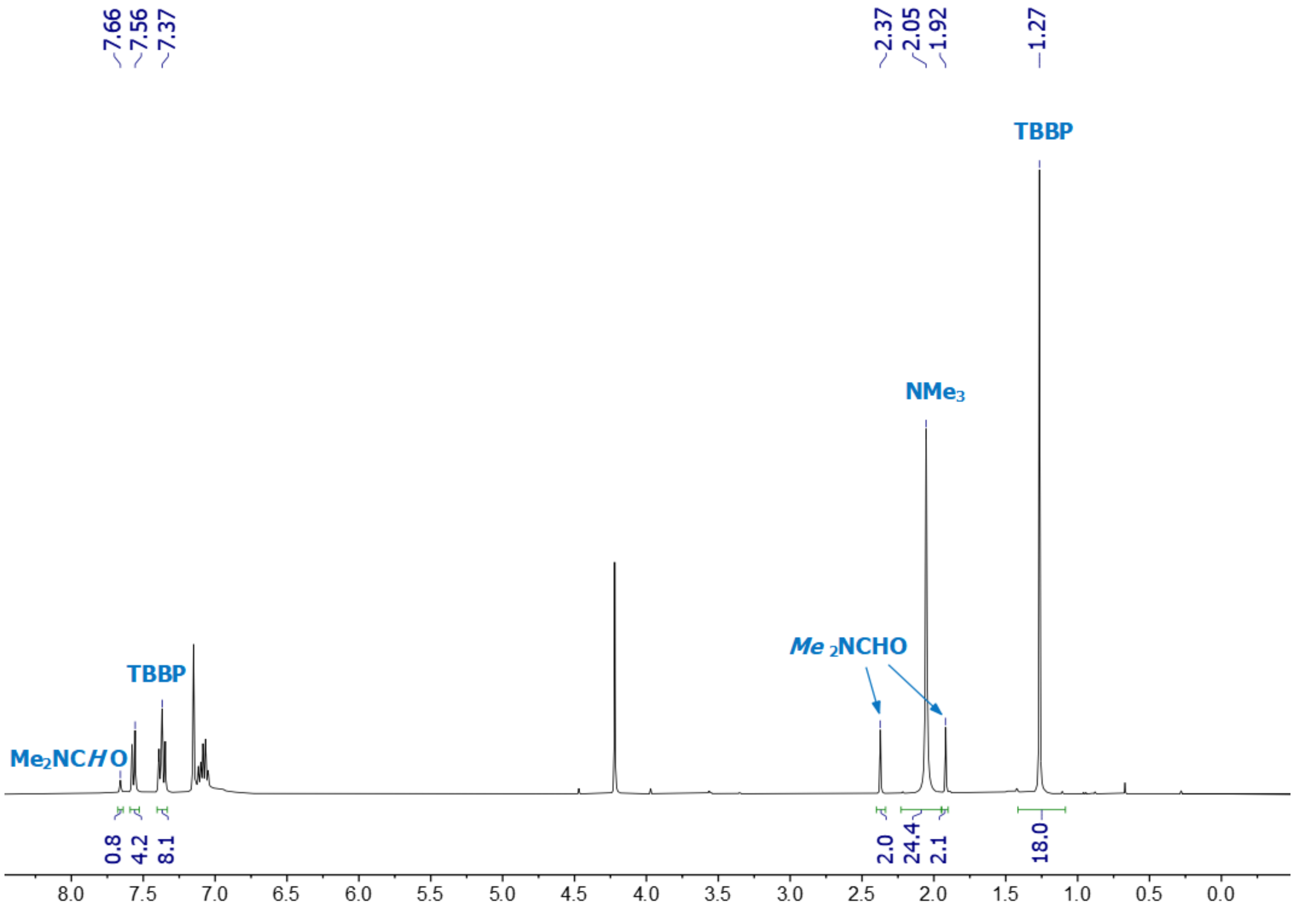

Fig. S23. ^{1}H NMR spectrum (400.2 MHz) of the catalytic reduction of DMF to Me_3N described above in the representative procedure using **1**·MeCN (5 mol%) and $PhSiH_3$ (1.5 eq) in C_6D_6. This is the bottom spectrum from the previous Figure with DMF, Me_3N and TBBP[a] peaks picked and integrated for quantification purpose.

Notes: Superimposition of some aromatic TBBP peaks with other aromatic peaks may cause integral discrepancy.

3. Crystallographic Data

Colour	Atom
	C
	O
	N
	F
	Si

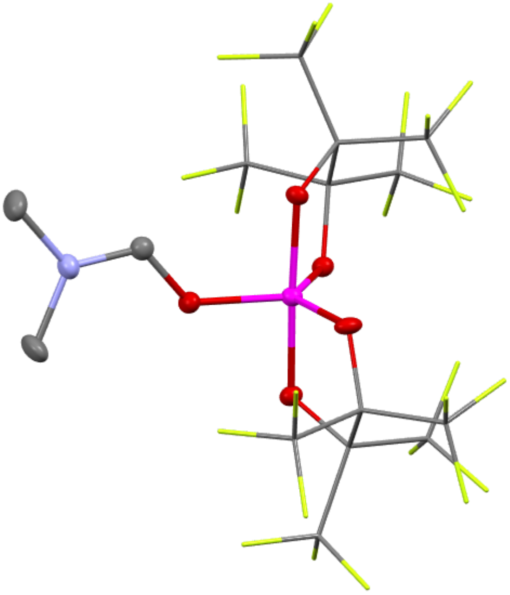

Fig. S24. Molecular structure of $(pin^F)_2Si\cdot Me_2NCHO$ (**1**$\cdot Me_2NCHO$) in the single crystal. Ellipsoids are shown at 50% probability level. Hydrogen atoms were omitted and $(C(CF_3)_2)_2$ scaffold shown as capped sticks for clarity. Crystallographic data Table S4 below.

Colour	Atom
	C
	O
	S
	F
	Si

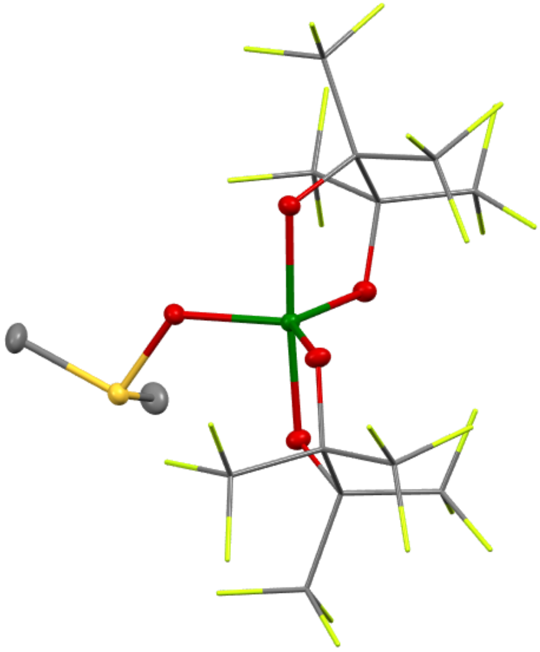

Fig S25. Molecular structure of $(pin^F)_2Ge\cdot Me_2SO$ (**2**$\cdot Me_2SO$) in the single crystal. Ellipsoids are shown at 50% probability level. Hydrogen atoms and one lattice MeCN omitted, and $(C(CF_3)_2)_2$ scaffold shown as capped sticks for clarity. Crystallographic data Table S4 below.

Table S4: Crystallographic Data Table for **1**·Me_2NHCO and **2**·Me_2SO.

	**1·Me_2NCHO**	**2·Me_2SO**
**CCDC number**	2492264	2492265
**Empirical formula**	$C_{15}H_7F_{24}NO_5Si$	$C_{14}H_6F_{24}GeO_5S \cdot CNCH_3$
**Formula weight**	765.31	855.89
**Temperature [K]**	100(2)	100(2)
**Crystal system**	monoclinic	monoclinic
**Space group (number)**	Pn (7)	$P2_1/n$ (14)
***a* [Å]**	7.7034(3)	10.9159(4)
***b* [Å]**	7.8823(4)	21.5040(7)
***c* [Å]**	19.5828(10)	11.1059(4)
**α [°]**	90	90
**β [°]**	92.096(2)	90.0560(10)
**γ [°]**	90	90
**Volume [Å^3]**	1188.28(10)	2606.95(16)
Z	2	4
**ρ_{calc} [gcm^{-3}]**	2.139	2.181
**μ [mm^{-1}]**	0.321	1.461
***F*(000)**	748	1664
**Crystal size [mm^3]**	0.062×0.156×0.231	0.176×0.202×0.246
**Crystal color**	colourless	colourless
**Crystal shape**	fragment	fragment
**Radiation**	MoK_α (λ=0.71073 Å)	MoK_α (λ=0.71073 Å)
**2θ range [°]**	4.16 to 51.52 (0.82 Å)	5.23 to 50.05 (0.84 Å)
**Index ranges**	−9 ≤ h ≤ 9<br>−9 ≤ k ≤ 9<br>−23 ≤ l ≤ 23	−12 ≤ h ≤ 12<br>−25 ≤ k ≤ 25<br>−13 ≤ l ≤ 13
**Reflections collected**	41117	40316
**Independent reflections**	4552<br>R_{int} = 0.0356<br>R_{sigma} = 0.0175	4598<br>R_{int} = 0.0314<br>R_{sigma} = 0.0196
**Completeness to θ = 25.242°**	100.0 %	99.8 %
**Data / Restraints / Parameters**	4552 / 2 / 417	4598 / 0 / 436
**Absorption correction T_{min}/T_{max} (method)**	0.7171 / 0.7453<br>(multi-scan)	0.6962 / 0.7453<br>(multi-scan)
**Goodness-of-fit on *F*^{2}**	1.156	1.049
**Final *R* indexes [$I \geq 2\sigma(I)$]**	R_1 = 0.0242<br>wR_2 = 0.0585	R_1 = 0.0177<br>wR_2 = 0.0455
**Final *R* indexes [all data]**	R_1 = 0.0259<br>wR_2 = 0.0604	R_1 = 0.0181<br>wR_2 = 0.0457
**Largest peak/hole [eÅ^{-3}]**	0.25/−0.27	0.37/−0.39

The data have been assigned the following deposition numbers which can either be quoted as CCDC Numbers or CSD Numbers. A CCDC Number is usually quoted for an organic or metal-organic structure, whereas a CSD Number is usually quoted for an inorganic structure.

CCDC XXXXXXX-YYYYYYY (generally used for organic and metal-organic structures)

CSD XXXXXXX-YYYYYYY (generally used for inorganic structures)

Deposition Number 2492264-2492265

Summary of Data - Deposition Number 2492264

Compound Name:
Data Block Name: data_mo_FroTo2_0m
Unit Cell Parameters: a 7.7034(3) b 7.8823(4) c 19.5828(10) Pn
----

Summary of Data - Deposition Number 2492265

Compound Name:
Data Block Name: data_FraDa315_0ma
Unit Cell Parameters: a 10.9159(4) b 21.5040(7) c 11.1059(4) P21/n
----

4. References

[S1] G. R. Fulmer, A. J. M. Miller, N. H. Sherden, H. E. Gottlieb, A. Nudelman, B. M. Stoltz, J. E. Bercaw and K. I. Goldberg, *Organometallics*, 2010, **29**, 2176–2179

[S2] Bruker, APEX suite of crystallographic software, Madison, Wisconsin, USA, 2015

[S3] Bruker, SAINT, Madison, Wisconsin, USA, 2016

[S4] Bruker, SADABS, Madison, Wisconsin, USA, 2016

[S5] G. M. Sheldrick, *Acta Crystallographica Section C: Crystal Structure Communications*, 2015, **71**, 3-8

[S6] G. M. Sheldrick, *Acta Crystallographica Section A: Foundations of Crystallography*, 2015, **71**, 3-8

[S7] C. B. Huebschle, G. M. Sheldrick, and B. Dittrich, *J. Appl. Cryst.*, 2011, **44**, 1281

[S8] G. M. Sheldrick, SHELXL-2014, Göttingen, Germany, 2014

[S9] A. J. C. Wilson and V. Geist, *International Tables for Crystallography. Volume C: Mathematical, Physical and Chemical Tables*, 1992, **C**, Tables 6.1.1.4 (pp. 500-502), 504.502.506.508 (pp. 219-222) and 504.502.504.502 (pp. 193-199)

[S10] A. L. Spek, PLATON, A Multipurpose Crystallographic Tool, Utrecht, Netherlands, 2010

[S11] C. F. Macrae, I. J. Bruni, J. A. Chisholm, P. R. Edgington, P. McCabe, E. Pidcock, L. Rodriquez-Monge, R. Taylor, J. van de Streek, and P. A. Wood, *J. Appl. Crystallogr.*, 2008, **41**, 466-470

[S12] D. Kratzert, FinalCif.

[S13] M. Muhr, P. Heiß, M. Schütz, R. Bühler, C. Gemel, M. H. Linden, H. B. Linden and R. A. Fischer, *Dalton Trans.*, 2021, **50**, 9031–9036.

[S14] F. S. Tschernuth, A. Kostenko, S. Stigler, A. Gradenegger and S. Inoue, *Dalton Trans.*, 2023, **53**, 74–81.

[S15] F. S. Tschernuth, T. Thorwart, L. Greb, F. Hanusch and S. Inoue, *Angew. Chem. Int. Ed.*, 2021, **60**, 25799–25803.
